# Author Correction: A solar-driven atmospheric water extractor for off-grid freshwater generation and irrigation

**DOI:** 10.1038/s41467-025-58181-y

**Published:** 2025-03-21

**Authors:** Kaijie Yang, Tingting Pan, Nadia Farhat, Alejandra Ibarra Felix, Rebekah E. Waller, Pei-Ying Hong, Johannes S. Vrouwenvelder, Qiaoqiang Gan, Yu Han

**Affiliations:** 1 Advanced Membranes and Porous Materials Center, Physical Sciences and Engineering Division, King Abdullah University of Science and Technology, Thuwal, 23955-6900 Saudi Arabia; 2 Sustainable Photonics Energy Research Lab, Material Science Engineering, Physical Science and Engineering Division, King Abdullah University of Science and Technology, Thuwal, 23955-6900 Saudi Arabia; 3 Water Desalination and Reuse Center, Division of Biological Sciences and Engineering, King Abdullah University of Science and Technology, Thuwal, 23955-6900 Saudi Arabia; 4 Center for Desert Agriculture, Division of Biological Sciences and Engineering, King Abdullah University of Science and Technology, Thuwal, 23955-6900 Saudi Arabia; 5 Center for Electron Microscopy, South China University of Technology, Guangzhou, 511442 China; 6 School of Emergent Soft Matter, South China University of Technology, Guangzhou, 511442 China

**Keywords:** Environmental chemistry, Solar thermal energy

Correction to: *Nature Communications* 10.1038/s41467-024-50715-0, published online 24 July 2024

In this article the authors name Nadia Farhat was incorrectly given as Nadia Ferhat. The original article has been corrected.

