# Supplementary material for: A solar-driven atmospheric water extractor for off-grid freshwater generation and irrigation
Source: Nat Commun. 2024 Jul 24;15:6260. doi: 10.1038/s41467-024-50715-0 (PMC11269568; doi:10.1038/s41467-024-50715-0)
Supplement: Supplementary file 1 — Supplementary Information [file 41467_2024_50715_MOESM1_ESM.pdf]

Supplementary information for

A solar-driven atmospheric water extractor for off-grid freshwater generation and irrigation

Kaijie Yang [a,b,c,1], Tingting Pan [a,1], Nadia Ferhat [c], Alejandra Ibarra Felix [c], Rebekah E. Waller [d], Pei-Ying Hong [c], Johannes S. Vrouwenvelder [c], Qiaoqiang Gan [b,c*] and Yu Han [a,e,f*]

[a] Advanced Membranes and Porous Materials Center, Physical Sciences and Engineering Division, King Abdullah University of Science and Technology, Thuwal, 23955-6900, Saudi Arabia

[b] Sustainable Photonics Energy Research Lab, Material Science Engineering, Physical Science and Engineering Division, King Abdullah University of Science and Technology, Thuwal, 23955-6900, Saudi Arabia

[c] Water Desalination and Reuse Center, Division of Biological Sciences and Engineering, King Abdullah University of Science and Technology, Thuwal, 23955-6900, Saudi Arabia

[d] Center for Desert Agriculture, Division of Biological Sciences and Engineering, King Abdullah University of Science and Technology, Thuwal, 23955-6900, Saudi Arabia

[e] Center for Electron Microscopy, South China University of Technology, Guangzhou 511442, China

[f] School of Emergent Soft Matter, South China University of Technology, Guangzhou 511442, China

[1] These two authors contributed equally.

[*] Corresponding authors: qiaoqiang.gan@kaust.edu.sa (Q. Gan) and yu.han@kaust.edu.sa (Y. Han)

Supplementary Notes 1

MTBs structure optimization

Vapor generation zone optimization

When the prototype be exposed to solar radiation, the solar absorber converts the incoming light into heat energy, which is then conducted through the structure of the MTBs. This leads to the formation of a temperature gradient, which in turn divides the MTBs structure into two distinct functional zones: a vapor generation zone with high temperature and an atmospheric water capture zone with room temperature (Supplementary Fig. 4). We characterized the temperature distribution of the MTBs structure during operation by IR imaging (Supplementary Fig. 5) and thermocouples (Supplementary Fig. 6), respectively. Once the distance to the top solar absorber reached approximately 3 cm, the temperature stabilized to be consistent with the room temperature, suggesting that the photothermal energy was primarily confined to the range above 3 cm. To investigate the relationship between temperature gradient and the configuration of vapor generation zone heights (H_v), we fabricated prototypes with varying H_v ranging from 0 to 4 cm (Supplementary Fig. 7) and evaluated their water production rate under 1 sun illumination (Supplementary Fig. 8). As shown in Supplementary Fig. 9, increasing the H_v from 0 to 3 cm resulted in a significant improvement in the water production rate from 0.18 to 0.36 L m^{-2} h^{-1}, indicating an increase in the heat utilization for vapor generation. Upon increasing the H_v from 3 to 4 cm, the production of water experienced a slight decrease. This outcome can be attributed to the fact that the MTBs region at room temperature inside the chamber tends to absorb the generated vapor, leading to a reduction in water production. These findings strongly suggest that an H_v of 3 cm represents the optimum value for achieving maximum water production performance. Furthermore, the stability of water production performance is closely associated with the occurrence of sorbent backflow.

GFM sheets number optimization

In the water production process, the liquid sorbent present in the vapor generation zone undergoes a gradual concentration. Driven by the concentration gradient and the density difference, concentrated sorbent will transport downward from the vapor generation zone to the atmospheric water capture zone via diffusion and convection, and its mass flow (J) can be described by the diffusion-convection equation:

$$J = J_{diff} + J_{conv} = nA\varepsilon(k_d \nabla C + k_c \nabla \rho) \quad (1)$$

where J_{diff} and J_{conv} present the mass flow rates caused by diffusion and convection, respectively. n is the GFM sheets number of the MTBs structure. A and ε are the cross-section area and the porosity of the GFM, respectively. k_d and k_c present the diffusion and convection coefficient of the sorbent. And, ∇C and $\nabla \rho$ present the concentration difference and density difference between the vapor generation zone and the atmospheric water capture zone.

As described by the equation, the mass flow of sorbent is proportional to the GFM sheets number (n). To investigate the relationships, we fabricated prototypes with n values ranging from 2 to 40 (Supplementary Fig. 10) and evaluated the performance stability by recording the evaporation rate variation (Supplementary Fig. 11). Due to the continuous concentration of LiCl on the evaporation surface, the evaporation rate of the prototype with 2 GFM sheets decreased gradually during the operation (Supplementary Fig. 12). Increasing the value of n demonstrated a noticeable effect on mitigating the rate of evaporation decay. As soon as n reached 32, the evaporation rate stabilized at approximately 0.78 kg m^{-2} h^{-1}, indicating an efficient sorbent backflow. However, increasing n from 32 to 40 led to only a marginal improvement in the evaporation rate. Hence, we ultimately optimized the n value to 32. To provide a visual representation of the efficient sorbents backflow, we conducted a supplementary experiment where the MTBs structure (n=32) was placed in deionized water and LiCl salt (approximately 1.5 g) was loaded onto the top of the structure (as shown in the experimental setup in Supplementary Fig. 13a). It is worth mentioning that magnetic stirring was used to eliminate concentration difference in the bulk water, ensuring precise conductivity detection. As demonstrated in Supplementary Fig. 13b, the loaded LiCl vanished within approximately 5 hours and the conductivity of the bulk water subsequently increased. These results strongly suggest that the sorbents can be effectively transported downward through the MTBs structure. To further demonstrate the salt backflow in LiCl solution without the stirring, we conducted another experiment. In this experiment, the MTBs structure was placed in 0.24 g g^{-1} LiCl solution, ~ 1.5 g LiCl was loaded on its top and the magnetic bar was removed from the system. As shown in Supplementary Fig. 15, without stirring, the loaded LiCl crystal gradually back flowed and completely dissolved after ~8 hours, confirming the efficient sorbent downward transport in LiCl solution without stirring. In our SAWE system, once the excess LiCl is returned to the atmospheric water capture zone that is exposed to the environment, it proceeds to capture

atmospheric water again.

Atmospheric water capture zone optimization

The use of MTBs structure effectively increases the contact area between the sorbent and the air, resulting in a significant enhancement in atmospheric water capture kinetics. This approach effectively overcomes the sluggish and slow kinetics typically observed in bulk LiCl solutions. We fabricated several prototypes with the height of the atmospheric water capture zone (H_a) ranging from 1 to 9 cm (Supplementary Fig. 16) and evaluated the water capture rates at 60% RH (Supplementary Fig. 17). As demonstrated in Supplementary Fig. 18, the water capture rate is proportional to the H_a, which significantly increased from 0.6 kg m^{-2} h^{-1} at the height of 1 cm to ~6.2 kg m^{-2} h^{-1} at the height of 9 cm. Using a representative H_a value of 5 cm, we further investigated the correlation between the water capture rate and the LiCl concentration under different RH conditions (Supplementary Fig. 19). As shown in Supplementary Fig. 20, with same LiCl concentration, the water capture rate at higher RH conditions (e.g. 90% RH) was observed to be significantly faster than that at lower RH conditions (e.g. 30% RH), which is attributed to the higher water content in the air under higher RH condition. In particular, at 90% RH, the water capture rate exhibited an increase from ~0.19 kg m^{-2} h^{-1} to ~3.63 kg m^{-2} h^{-1} as the LiCl concentration was raised from 0.10 g g^{-1} to 0.45 g g^{-1}. This phenomenon can be attributed to the fact that a more concentrated LiCl solution has greater number of sites to capture the water molecules.

In addition to the water capture rates, we also examined the relationship between the water production rate and the LiCl concentration. Interestingly, we observed an inverse correlation between the two, whereby the water production rate decreased from ~0.78 kg m^{-2} $h^{-1}$to ~0.05 kg m^{-2} h^{-1} as the LiCl concentration increased from 0.13 g g^{-1} to 0.34 g g^{-1} (Supplementary Fig. 20**)**. Given that the water capture rate generally surpasses the water production rate under most circumstances when the H_a is set at 5 cm, we selected a fixed H_a value of 5 cm for the subsequent performance evaluations.

Supplementary Notes 2

Sorbent distribution characterization

The system will reach an equilibrium state after a certain period of operation. In this equilibrium state, the LiCl concentration in the MTB structure decreases from the top, forming a concentration gradient that drives the transport of sorbent. To determine the salt concentration gradient in the equilibrium states under different RH conditions, we carefully analyzed the salt content distribution along the MTB structure.

In this experiment, we extracted the GFMs from a system in a given equilibrium condition (Supplementary Fig. 21a), cut the GFM into strips with a width of 0.5 cm (Supplementary Fig. 21b), and placed the strips in 10 ml of deionized water to dissolve the contained salt (Supplementary Fig. 21c). According to the relationship between the salt concentration and the solution conductivity (Supplementary Fig. 22), the salt content of each strip was calculated by the equation: $\mathrm{c} = \frac{(m_w+m_g-m_d)\times c_c}{(m_g-m_d)}$, where m_w is the deionized water amount (i.e. 10 g), m_g is the weight of the wet GFM strip, m_d is the weight of the 0.5 cm width GFM strip (i.e. 0.033 g), c_c is the solution concentration determined by the conductivity. As shown in Supplementary Fig. 23a, the equilibrium concentration at high RH is higher than that at low RH. And, the salt concentration gradually decreases along the GFM and stabilizes at a distance of approximately 4 cm from the top. To visually demonstrate the salt migration, we depicted the LiCl distribution map according to the measured salt content (Supplementary Fig. 23b).

Supplementary Notes 3

Solar-water collection efficiency calculation

The solar-water collection efficiency at the equilibrium state can be calculated using the following equation:

$$\eta_s = \frac{(h_{lv}+h_d)\times m_{cw}}{A_{sa}\times q_{sol}\times t}$$

where η_s presents the solar-water collection efficiency, h_{lv} is the evaporation enthalpy of pure water, h_d is the differential enthalpy of dilution, m_{cw} is the collected water amount, A_{sa} is the size of the solar absorber, q_{sol} is the solar flux and t is the operation time.

According to the equilibrium concentration of the LiCl (Supplementary Fig. 23) and its corresponding h_d (Supplementary Fig. 25), we can calculate the solar-water collection efficiency at different RH levels.

Water production estimation

The estimation of water production in Jeddah, Saudi Arabia was calculated by considering the solar-water collection efficiency across different RH conditions, alongside the daily average humidity and solar irradiation data for Jeddah in 2022. The environment data was sourced from weather website *Time and Date* (https://www.timeanddate.com/weather/saudi-arabia/jeddah/historic?month=1&year=2022).

The water production estimation on a global scale was derived by incorporating the solar-water collection efficiency across varying RH conditions, along with the yearly average humidity and solar irradiation data. The global yearly average humidity and solar irradiation date were cited from *Climate Research Unit* (https://sage.nelson.wisc.edu/data-and-models/atlas-of-the-biosphere/mapping-the-biosphere/ecosystems/average-annual-relative-humidity/) and *Global horizontal irradiation* (https://globalsolaratlas.info/download/world)

Supplementary Notes 4

Irrigable area estimation

The water requirements for plant growth are primarily determined by climate conditions, specific plant species, and the intended purpose of cultivation. In this study, we selected two distinct plant growth systems for evaluation: a cropping system featuring Chinese cabbage and a desert tree plantation involving *Vachellia tortilis* (also known as *Acacia tortilis*).

Agricultural irrigation must be precise in order to achieve maximum yields per square meter, whereas landscape irrigation especially for desert species is more flexible since these plants can survive with limited water input for extended periods of time. The daily water requirement for Chinese cabbage is estimated to be 2.50 - 4.16 L m^{-2} per day and the water requirement for each *Vachellia tortilis* is 0.14 - 1.3 L per day (Supplementary Table 4). Based on the water production capacity of our system (Supplementary Fig. 29) and the water requirement for plant growth, we depicted the irrigable capability of our system for Chinese cabbage (Supplementary Fig. 45a) and *Vachellia tortilis* (Supplementary Fig. 45b) at different RH conditions. Specifically, at 70% RH, 1 m^2 system can sustain the growth of up to ~ 0.8 m^2 Chinese cabbage in maintenance-free mode and ~1.0 m^2 Chinese cabbage in manual mode. Meanwhile, for desert plantation, at 70% RH, 1 m^2 system can sustain the growth of more than 3 *Vachellia tortilis* in maintenance-free mode and over 4 *Vachellia tortilis* in manual mode. It's worth noting that the system's irrigation capacity will continue to improve as the relative humidity (RH) increases. Additionally, for certain high-water-demand plants, while the system may not fully meet their water requirements, it can significantly reduce their dependence on liquid water resources by harvesting water from the air.

Supplementary Notes 5

Thermal conductivity evaluation

We measured the thermal conductivity of the solar absorber and the glass fiber membrane (GFM) containing 0.24 g g^{-1} LiCl solution using thermal conductivity meter (TPS 2500 S, Hot Disk). The measured thermal conductivity of the solar absorber and the GFM was ~0.051 W mK^{-1} and 0.42 W mK^{-1}, respectively.

Supplementary Notes 6

Cost analysis

The materials used for our system fabrication includes: glass fiber membrane, carbon nanotube and acrylic plate. There cost are listed below:

- Glass fiber membrane: 0.41 USD per square meter.
- Carbon nanotube: 2.9 USD per gram.
- Acrylic plate: 4.7 USD per square meter.

Assuming that a 1 m^2 system needs ~ 50 m^2 glass fiber membrane, ~5 g carbon nanotubes, and ~4 m^2 acrylic plate, a stand-along system will cost ~54.8 USD. By considering more cost-effective materials, such as nonwoven fabric and carbon black can be employed to replace the glass fiber membranes and carbon nanotube, the manufacturing cost can be further reduced to ~23 USD per 1-m^2 system.

Supplementary Notes 7

Qi et al. introduced a system for atmospheric water harvesting characterized by simultaneous adsorption-desorption capabilities[8]. This pioneering system represented a significant advance in the field, it primarily focused on interfacial evaporation and the use of an exceptional hygroscopic sorbent, namely [EMIM][Ac]. Compared with that pioneering system, our design introduced efficient mass transport and optimized heat management, and efficiently minimized the footprint of the system. When using same liquid sorbent, our system demonstrated a substantial performance improvements.

In order to highlight the inherent advantages of our architectural design, we reproduced the prior system detailed in ref. 8 (Supplementary Fig. 48a) and conducted a rigorous evaluation under identical conditions, where the system's dimensions were meticulously tailored in accordance with the size specifications provided in the referenced literature (Supplementary Fig. 48b). Specifically, this evaluation was carried out consistently at 70% RH and 25°C, utilizing a LiCl solution with an initial concentration of 0.24 g g^{-1} as the liquid sorbent. The evaluation lasted 8 days, with 8 hours under 1 sun illumination and 16 hours under dark environment. As illustrated in Supplementary Fig. 49, normalized by the footprint of the system, the water production of our system is around 9.6 times higher than the pioneering system reported in ref. 8. This striking difference demonstrates a remarkable improvement in water productivity, attributable to the efficient heat energy utilization and meticulous optimization of architecture that significantly reduce the system's physical footprint. To provide additional clarity, when aiming for a target water productivity of 5 kg per day, our system would necessitate a mere 2.6 m^2, whereas the control system would require a much more substantial 25 m^2. This underscores the unmistakable advantage of our innovative design, not only in terms of performance but also in optimizing space efficiency. These attributes are of paramount importance for the practical implementation of such an AWE device, as they maximize its potential impact on sustainability efforts.

Supplementary Methods

Prototype fabrication

The solar absorber was fabricated by loading partially oxidized carbon nanotubes (CNTs) onto the glass fiber membrane (GFM) with a controlled loading percentage ~10 wt.%. We selected a CNT-loaded GFM with a loading percentage of ~10 wt.% CNT as the solar absorber material because it ensures

comprehensive coverage of GFM with CNTs, resulting in high solar absorptance (Supplementary Fig. 47). The mass transport bridges (MTBs) structure was then created by assembling the GFM (~0.45 mm thick with ~60% porosity) into the designed PMMA frame. Specifically, GFM was firstly cut into strips with a width of 3 cm, and the PMMA frame was fabricated via laser cutting and reassembling of the units. Then, these strips were assembled into the designed PMMA frame to realize the MTBs structure fabrication. The final prototype was constructed by combing the MTBs structure, the solar absorber, the condenser chamber and the container together and infiltrating the LiCl solution into the MTBs structure (see details in Supplementary Fig. 3). In this study, GFM was purchased from a commercial supplier (i.e., Haining Taoyuan Chemical Instrument Factory, with a product model of 49# glass fiber membrane). The CNTs were acquired from Sigma-Aldrich, with specifications indicating a diameter of 110-170 nm and a length of 5-9 μm. LiCl was obtained from VWR Chemicals with purity > 99%. It is worth noting that the solar absorber and the MTBs structure were in close contact. Furthermore, during operation, both the solar absorber and the MTBs are fully saturated with a LiCl solution, which renders the structure flexible and promotes a secure attachment. This close contact ensures efficient heat and mass transport between these two structures.

Performance evaluation

The performances of prototypes were evaluated in a research chamber (AR66L, Percival) where the temperature and RH can be controlled accurately. The evaluations were carried out at 60% RH, 25℃ with the MTBs structure of the prototypes initially infiltrated with 0.24 g g^{-1} LiCl solution. For water production performance evaluation, the vapor generation zone was enclosed in the condenser chamber and the water production rate was determined by measuring the collected water. For evaporation performance evaluation, the condenser chamber was removed to allow for vapor release, the atmospheric water capture zone was sealed to avoid the effect of water capture, and the evaporation rate was determined by detecting the weight change. For water capture performance evaluation, the atmospheric water capture zone was exposed to the environment while the vapor generation zone was enclosed, and the water uptake rate was determined by measuring the weight change. To evaluate the performance of water capture and water production at different RH and different LiCl concentration, the environment RH and the infiltrated LiCl solution concentration was regulated accordingly.

The experiment, which involved recording the system's operational process using time-lapse

photography, was conducted at 90% RH, 25℃ with MTBs structure initially infiltrated with 0.24 g g^{-1}LiCl solution. The prototype underwent 12 h of water capture without sunlight, followed by 12 h of water production under 1 sun illumination.

The simultaneous water capture and production performance was evaluated at 65% RH, 25℃ with MTBs structure initially infiltrated with 0.24 g g^{-1} LiCl solution. The water capture performance was determined by recording the weight change of the prototype and the water production was measured by weighting the condensed water.

The day-by-day cycling evaluation was performed to evaluate the water production potential under different RH conditions. The MTBs structure of the prototype was infiltrated with 0.24 g g^{-1} LiCl and the temperature was controlled at 25°C. In each cycle, the prototype first underwent 16h of water capture followed by 8h water production under 1 sun illumination. The water production rate was calculated according to the condensed water in the chamber.

Field tests

The outdoor experiment was conducted on the rooftop in KAUST. The environmental conditions, including RH, temperature, and solar intensity were recorded by a weather station (HP2550, Misol). The solar energy was calculated according to the following equation: $\mathrm{E} = \mathrm{A} \times \int P(t)dt$ where E (kJ) represents the received solar energy, A (m^2) is the irradiated area, P (kW m^{-2}) is the solar intensity and t (s) is the time. The average daily temperature and humidity are calculated based on the real-time weather data between 19:00 yesterday to 19:00 today. The produced water was collected by a graduated cylinder and the process was recorded by a camera. The ions concentration was measured by ICP-OES (5110, Agilent). The total intact cell concentration was quantified by flow cytometry (BD Accuri C6, Belgium). HPC was determined using the Quanti-Tray method. Colilert-18 test kit (IDEXX, USA) was used to simultaneously detect total coliforms and E. coli in the water sample. The active biomass present in the water samples were evaluated with a luminometer (Celsis Advance Luminometer, Charles River Laboratories). Specifically, LuminEx and LuminATE-HS were used to release the ATP and produce light.

The off-grid irrigation experiment was performed on the balcony in KAUST. A custom-built acrylic tray with ten individual compartments (6×6×5 cm) was used for plant growth. Chinese cabbage seed (Quality Cabbage, Longda Seed) was selected for demonstration and standard potting soil

(Basissubstrat 2, Stender) was used for plant growth. The automatic drip irrigation was controlled by a timing pump which supplied 9 ml of water to each compartment daily at 20:00. The volumetric water content and the conductivity of the soil were detected by a soil detector (PR-3001-TRREC-N01, Presens) every night at 19:00. The probe was located 3 cm away from the roots to avoid any interference on the plant growth. The plant growth was recorded by a camera at 8:00. The plants height was measured with a ruler. The leaf number was accounted every 4 days. Specifically, the leaf size was determined by tracing its outline on a grid paper and calculating the area of the outline. We place the leaf on the grid paper with squares that are 0.25 cm^2 in area. Then, we traced the outline of the leaf on the grid paper and counted the number of the squares to get the surface of each leaf. The plants were harvested after 20 days of growth. The wet biomass of plants was recorded after cleaning the soil attached to the roots, and the dry biomass was determined following dehydration at 80℃ for 12h. The repetition experiment of plant irrigation was performed on the rooftop in KAUST. This repetition contains two sets of plant growth, one was irrigated with the collected atmospheric water and the other was irrigated with the tap water. The irrigation water volume for each plant (i.e. ~9 ml) and watering time (i.e. at 20:00 each day) were kept consistent with the previous experiment. The plant growth was recorded accordingly. The fresh weight and the dry weight of the harvested plants were analyzed.

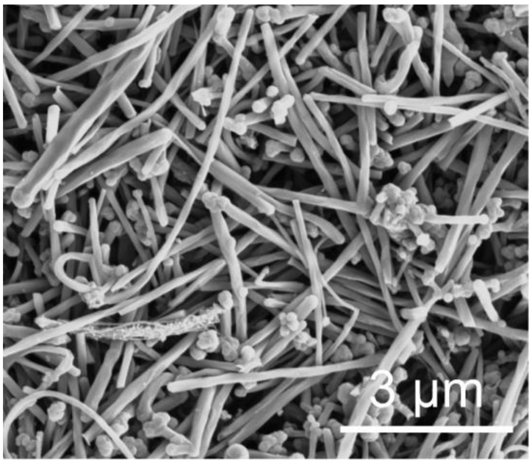

Supplementary Fig. 1| SEM image of the solar absorber.

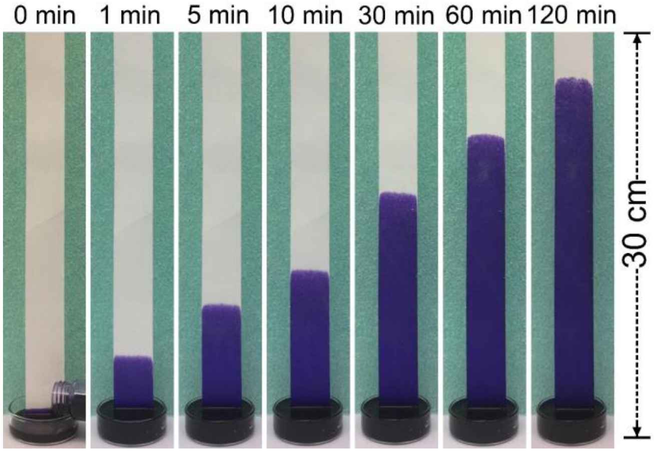

Supplementary Fig. 2| Capillary rise of water along the GFM. The water was dyed by methylene blue (MB) to enhance the visualization. During the capillary rise process, MB solution fills the porous spaces in the GFM, giving rise to a blue appearance that effectively illustrate the solution transport process.

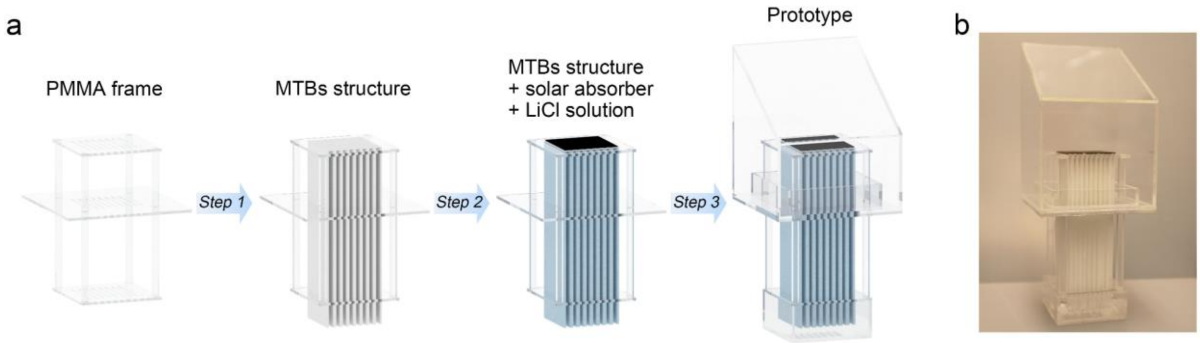

Supplementary Fig. 3| System fabrication. a, The schematic of the prototype assembly includes *Step 1*: GFMs assembly; *Step 2*: solar absorber assembly and LiCl solution infiltration; *Step 3*: chamber and container assembly. **b**, A photograph of the fabricated prototype (evaporation area: 3 cm ×3 cm).

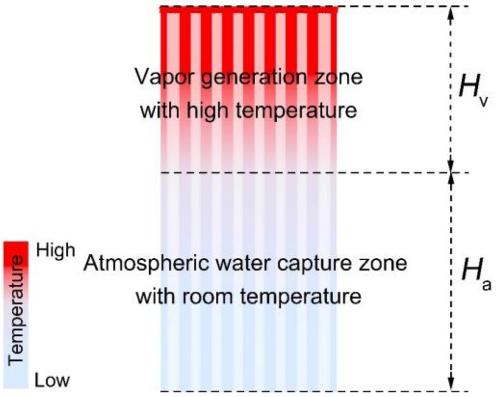

Supplementary Fig. 4| The demonstration of temperature gradient along the MTBs structure.

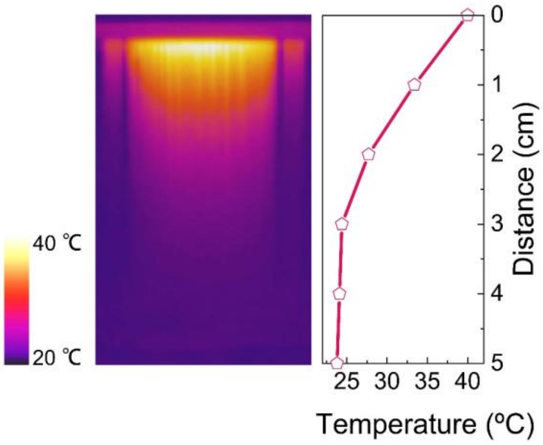

Supplementary Fig. 5| The IR image of the MTBs structure during operation, illustrating the temperature distribution. The experiment was performed under 1 sun illumination at ~21℃, 60% RH. The MTBs structure was infiltrated with 0.24 g g^{-1} LiCl solution.

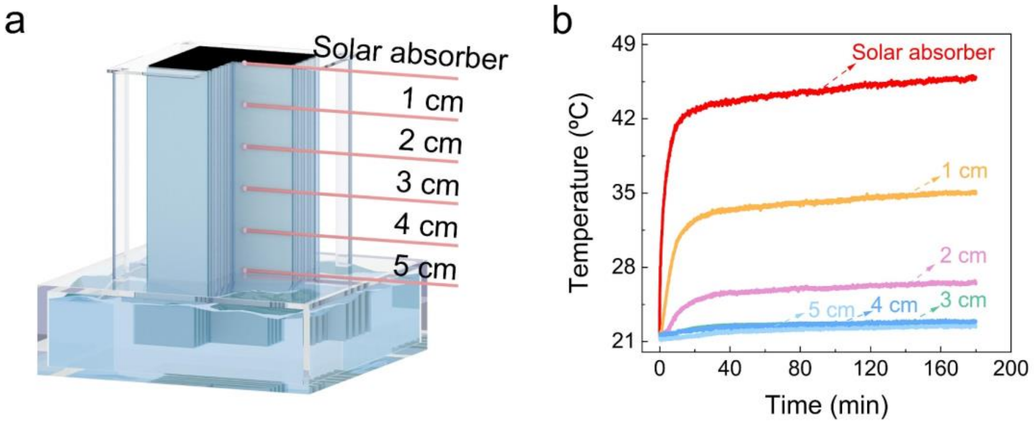

Supplementary Fig. 6| Temperature variation inside the MTBs structure. a, Experiment setup and the location of temperature test points. **b**, The temperature variations at the test points during operation. The experiment was performed under 1 sun illumination at ~21℃, 60% RH. The MTBs structure was infiltrated with 0.24 g g^{-1} LiCl solution.

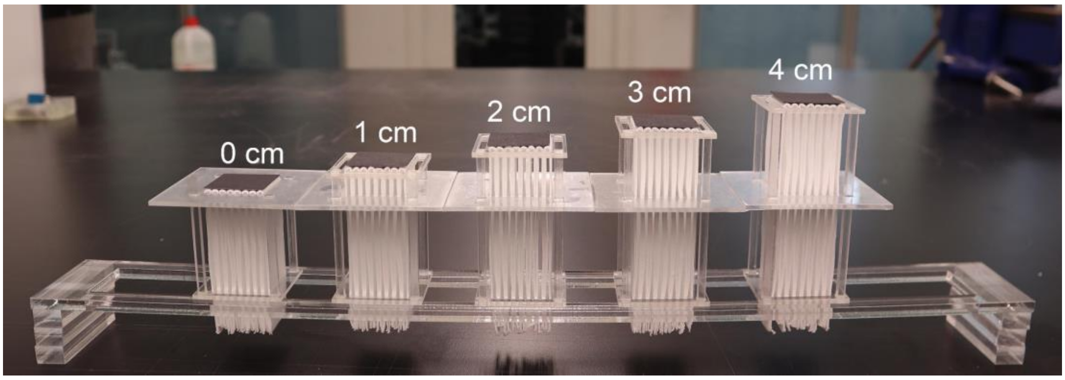

Supplementary Fig. 7| A photograph of prototypes with H_v ranging from 0 to 4 cm.

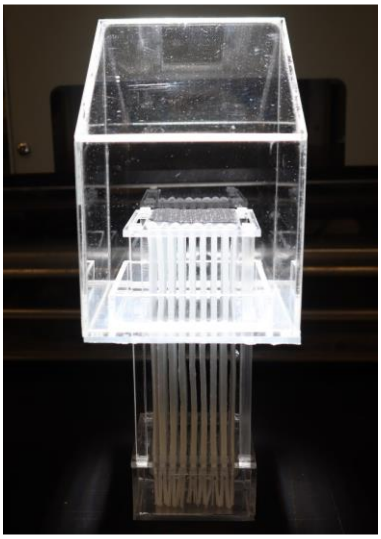

Supplementary Fig. 8| Experiment setup for water production evaluation. During the evaluation, the vapor generation zone is enclosed in the chamber for vapor condensation and water collection. The experiment was performed under 1 sun illumination at ~25℃, 60% RH. The MTBs structure was infiltrated with 0.24 g g g^{-1} LiCl solution.

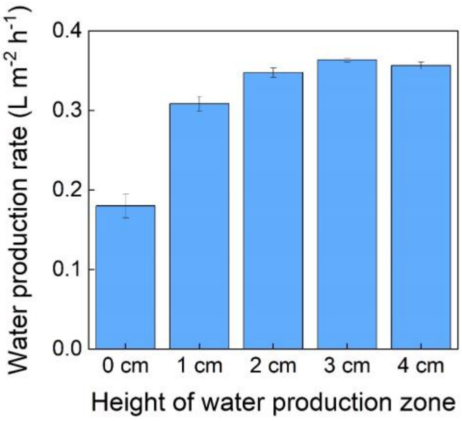

Supplementary Fig. 9| The relationship between the water production rate and the H_v. Error bar present the standard deviation between 2 evaluations.

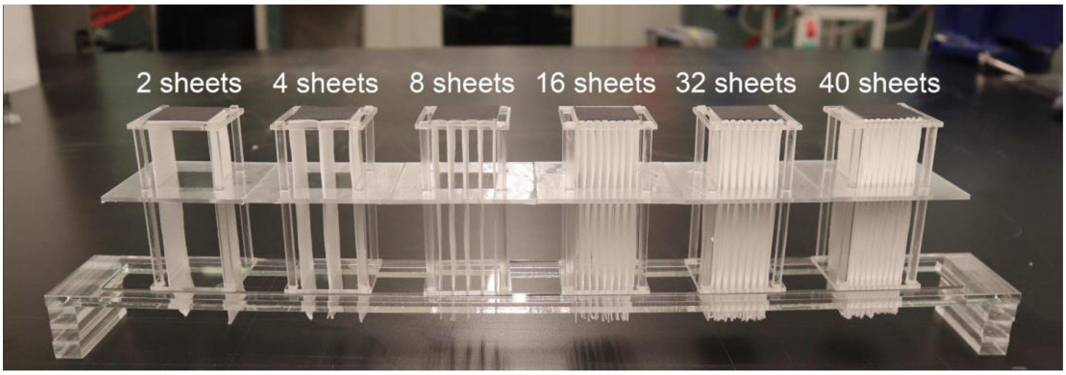

Supplementary Fig. 10| A photograph of prototypes with *n* ranging from 2 to 40. The H_v was set to 3 cm.

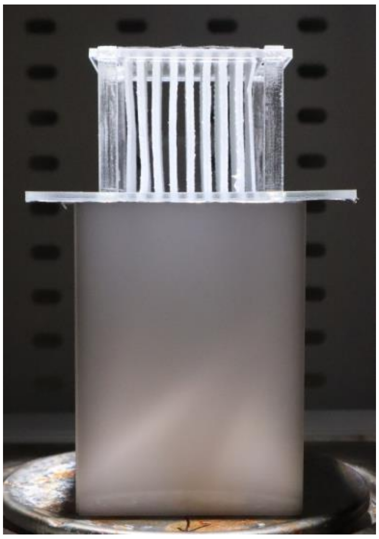

Supplementary Fig. 11| Experiment setup for evaporation performance evaluation. During the evaluation, the atmospheric water capture zone was sealed to avoid the influence of atmospheric water capture on weight change. The experiment was performed under 1 sun illumination at ~25℃, 60% RH. The MTBs structure was infiltrated with 0.24 g g^{-1} LiCl solution.

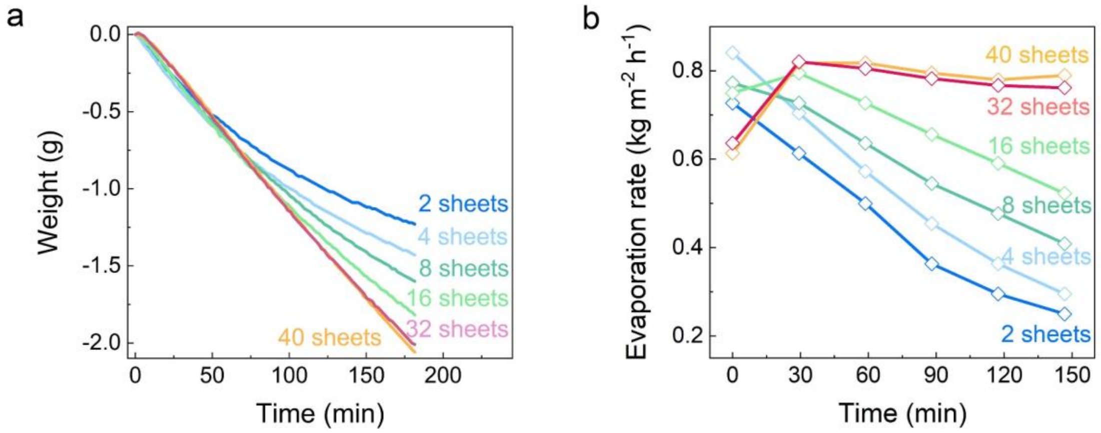

Supplementary Fig. 12| The vapor generation performance of MTBs structure with different *n*. a, Mass change curves of MTBs structures with different *n*. **b**, The evaporation rate variation of MTBs structures.

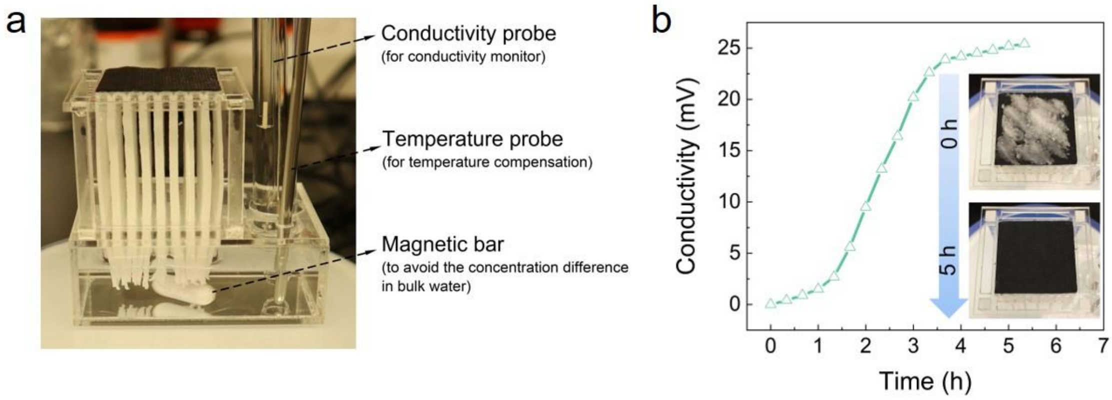

Supplementary Fig. 13| Salt backflow evaluation. a, Experiment setup for evaluation. **b**, The water conductivity variation and the photo of the MTBs structure surface. In this experiment, 3 cm high MTBs structure with 32 GFM sheets was used to present the vapor generation zone. ~1.5 g LiCl sorbent was placed on its top. The sorbent backflow process was recorded by time-lapse photography and the conductivity variation of the bulk water was monitored by a conductivity meter.

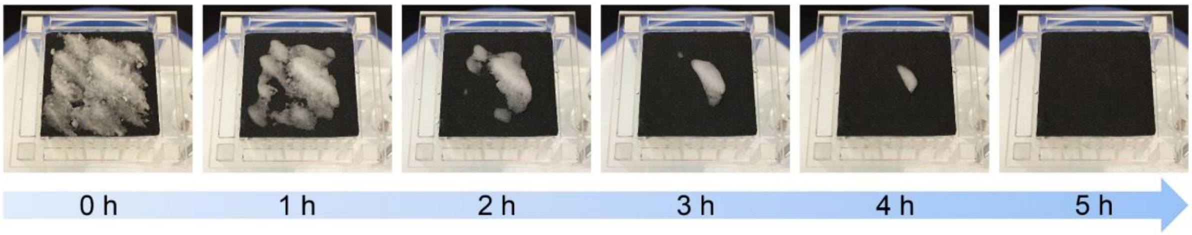

Supplementary Fig. 14| The recording of the sorbent backflow process.

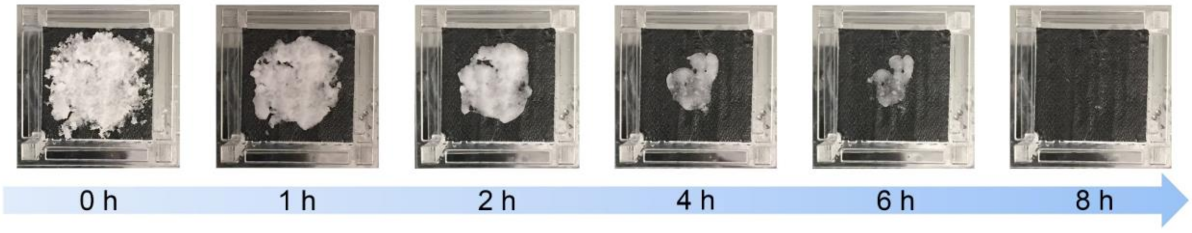

Supplementary Fig. 15| The observation of sorbent backflow in LiCl solution without stirring. In this experiment, the MTBs structure was placed in 0.24 g g^{-1} LiCl solution without magnetic stirring to demonstrate that the sorbent can efficiently backflow in LiCl solution without stirring.

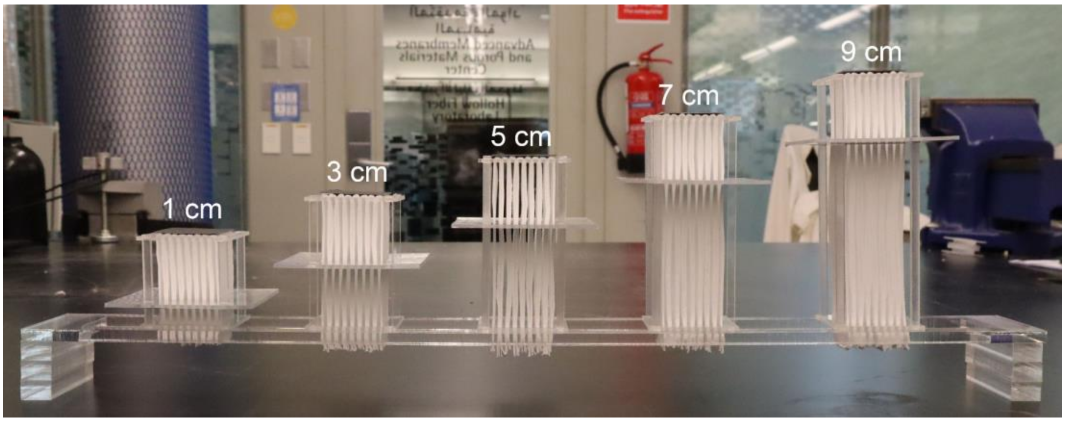

Supplementary Fig. 16| A photograph of prototypes with $\boldsymbol{H_a}$ ranging from 1 to 9 cm. The H_v was set to 3 cm.

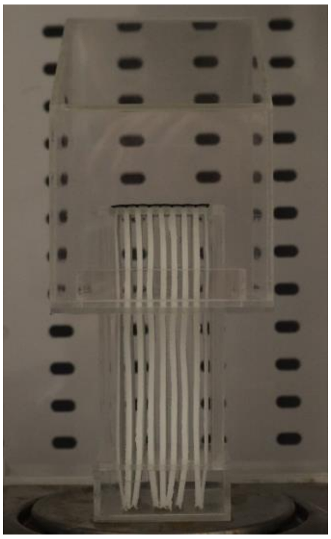

Supplementary Fig. 17| Experiment setup for atmospheric water capture evaluation. The experiment was performed in a climate chamber which can control the RH and temperature accurately.

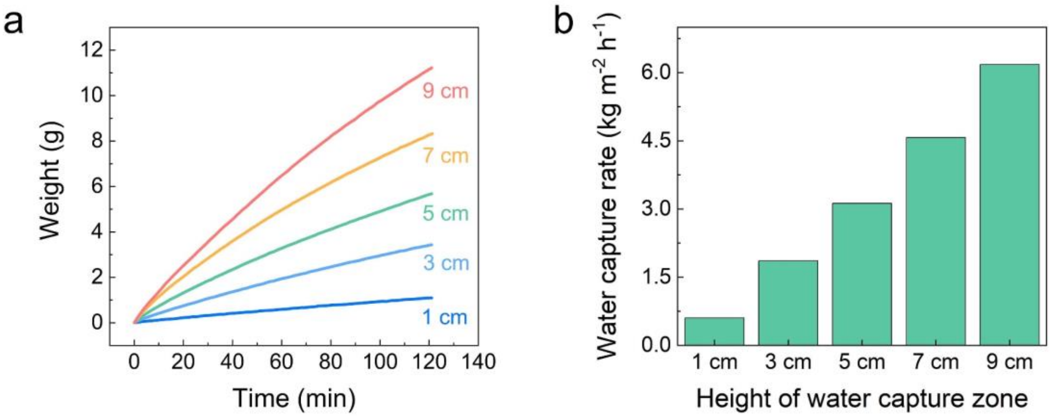

Supplementary Fig. 18| The water capture performance of prototypes with different $\boldsymbol{H_a}$. a, Mass change curves of prototypes with different H_a. **b**, The water capture rate of prototypes.

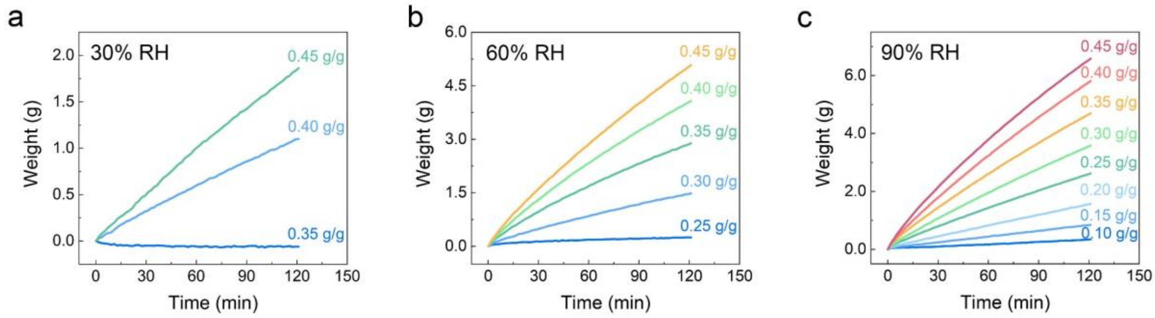

Supplementary Fig. 19| The water capture performance of prototypes infiltrated with LiCl solutions with varying concentrations. **a**, Weight change curves at 30% RH. **b**, Weight change curves at 60% RH. **c**, Weight change curves at 90% RH.

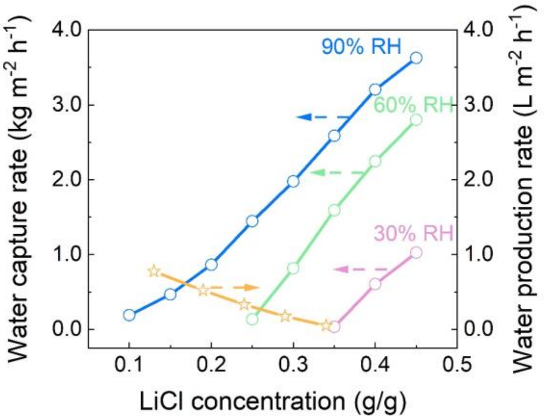

Supplementary Fig. 20| The water capture rate and water production rate of prototypes infiltrated with different LiCl solution concentrations under varying RH conditions. The blue, green and pink curves represent the water capture rates, while the yellow curve represents the water production rate.

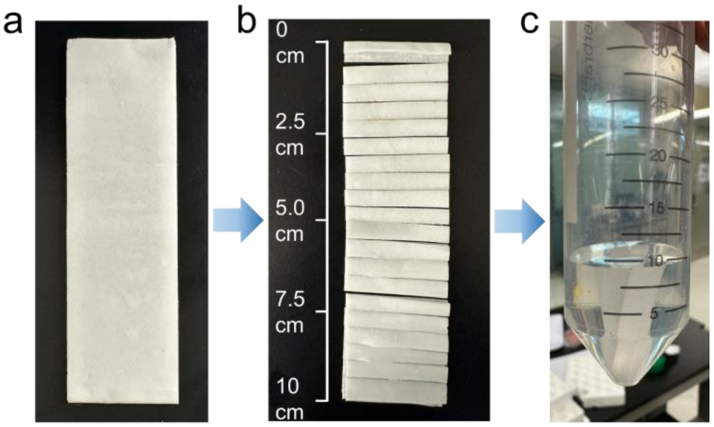

Supplementary Fig. 21| Experiment setup for salt distribution characterization. **a**, GFM extracted from the prototype that has reached the equilibrium at the given RH. **b**, Photo of strips cut into 0.5 cm width strips. **c**, Photo of the strip in the deionized water.

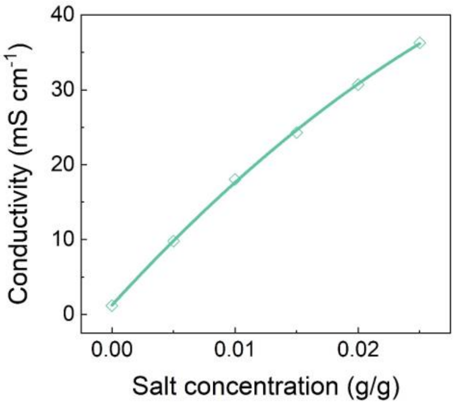

Supplementary Fig. 22| The relationship between the salt concentration and the conductivity.

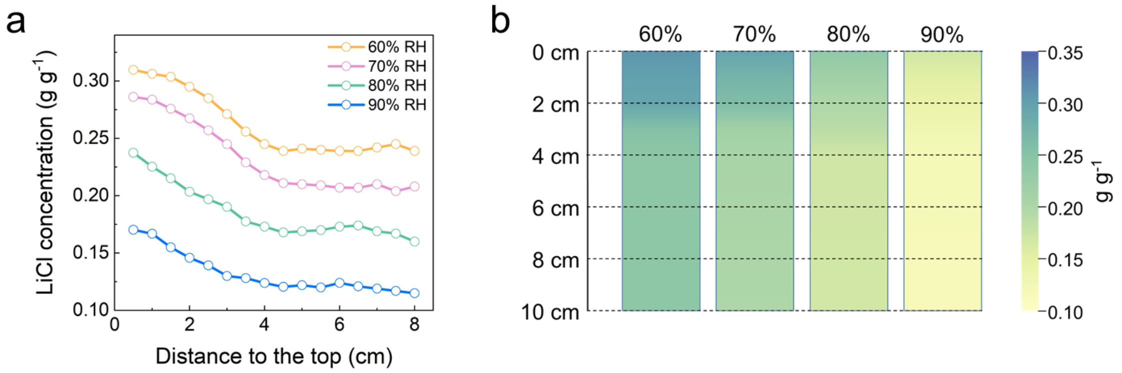

Supplementary Fig. 23| The sorbent content distribution along the MTBs structure. a, The LiCl concentration distribution along the GFM equilibrium at different RH. **b**, The visualization of salt concentration distribution along the MTBs structure.

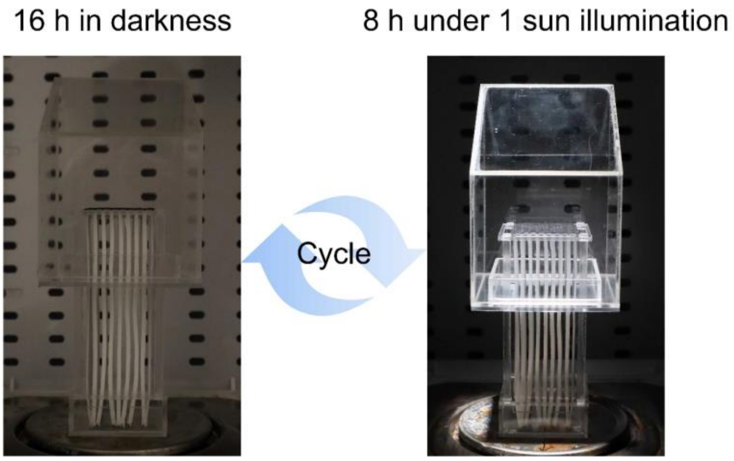

Supplementary Fig. 24| The demonstration of maintenance-free operation. In this operation mode, the system run independently and there is no need to open or close the chamber during the operation.

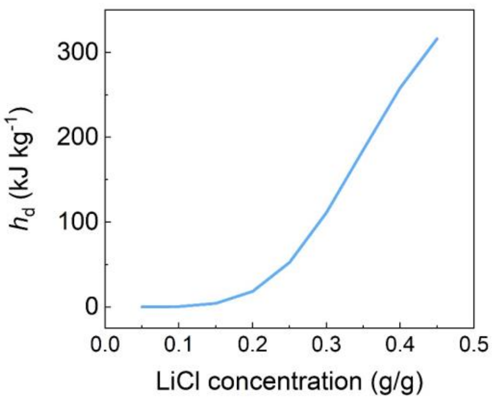

Supplementary Fig. 25| The curve of differential enthalpy of dilution according to the LiCl concentration (data from Ref. 1).

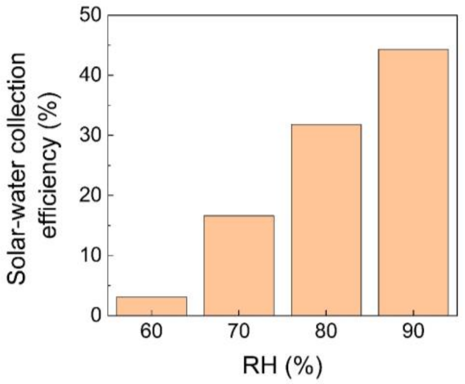

Supplementary Fig. 26| The solar-water collection efficiency of the system under different RH conditions. The efficiency of solar-water collection demonstrates a notable increase with rising RH. Specifically, it is computed to be 3.1%, 16.6%, 31.8%, and 44.3% at RH levels of 60%, 70%, 80%, and 90%, respectively. To put this into perspective, to generate 1 liter of water, the system will consume solar energy amounts of 22.7 kWh, 4.2 kWh, 2.2 kWh, and 1.5 kWh at 60% RH, 70% RH, 80% RH, and 90% RH, respectively.

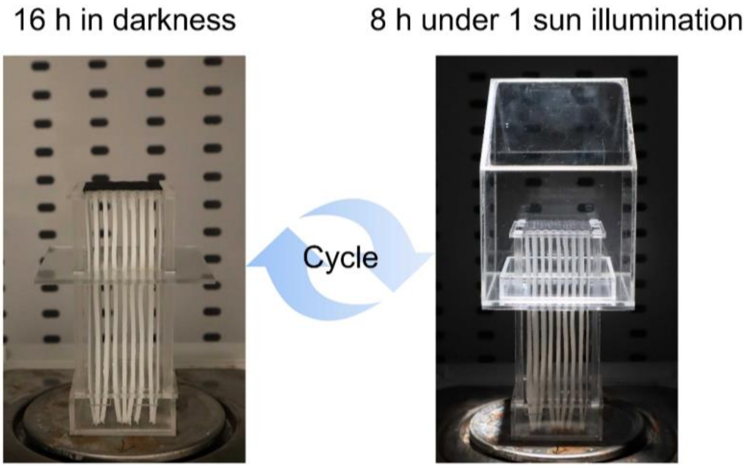

Supplementary Fig. 27| The demonstration of manual mode. In this operation mode, the chamber is removed during the atmospheric water capture process in darkness. While, during the water production process, the chamber was added to condense the generated vapor and collect the condensed water. Thereby, manual operation is required to switch the water capture and water generation process.

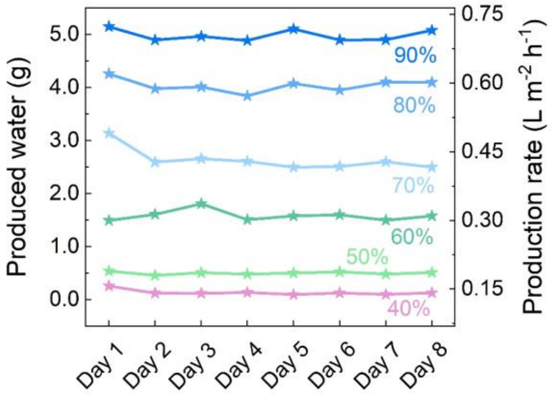

Supplementary Fig. 28| The water production performance of the system operated in manual mode.

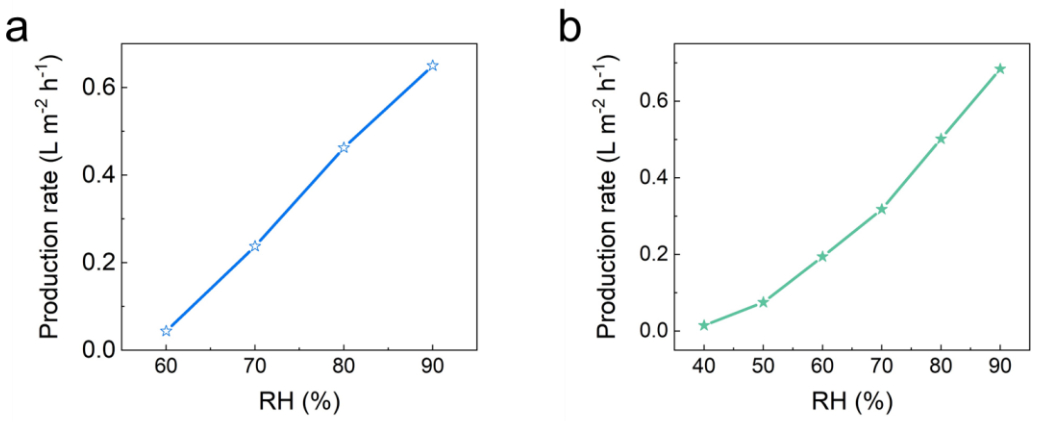

Supplementary Fig. 29| Correlation between the water production rate and the RH. a, The relationship between water production rate and the RH in maintenance-free mode. **b**, The relationship between water production rate and the RH in manual mode.

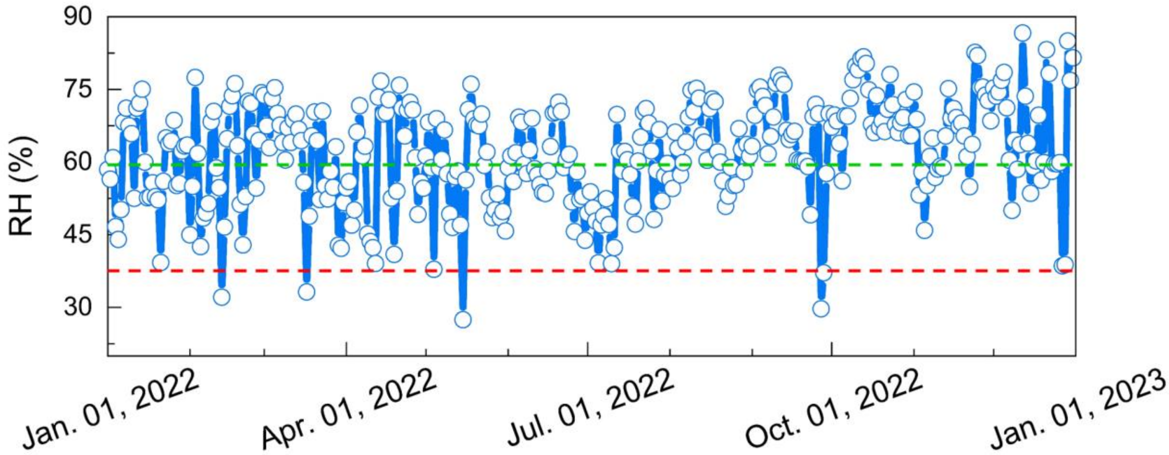

Supplementary Fig. 30| The daily average RH variation of Jeddah, Saudi Arabia throughout the year of 2022. The green and red dash line presents the applicable limit of system operated in maintenance-free mode and the manual mode, respectively.

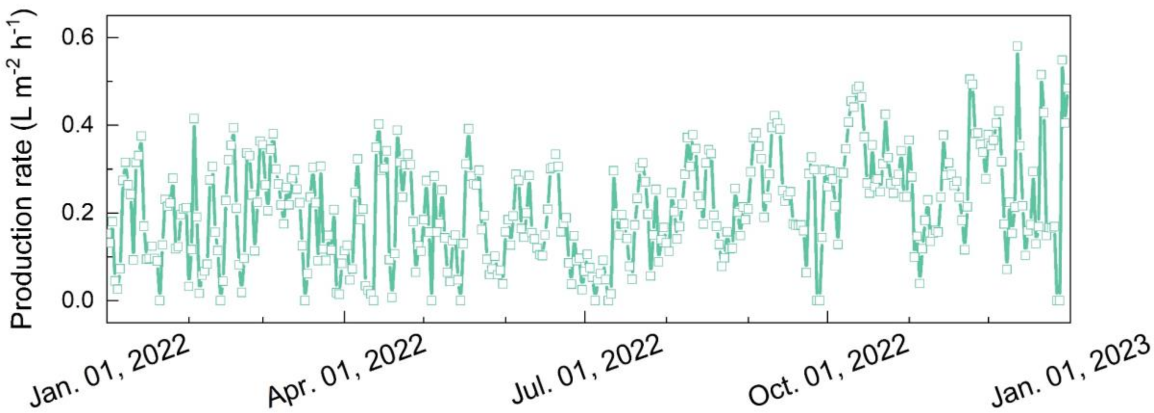

Supplementary Fig. 31| The estimated water production rate of our system in Jeddah operated in manual mode.

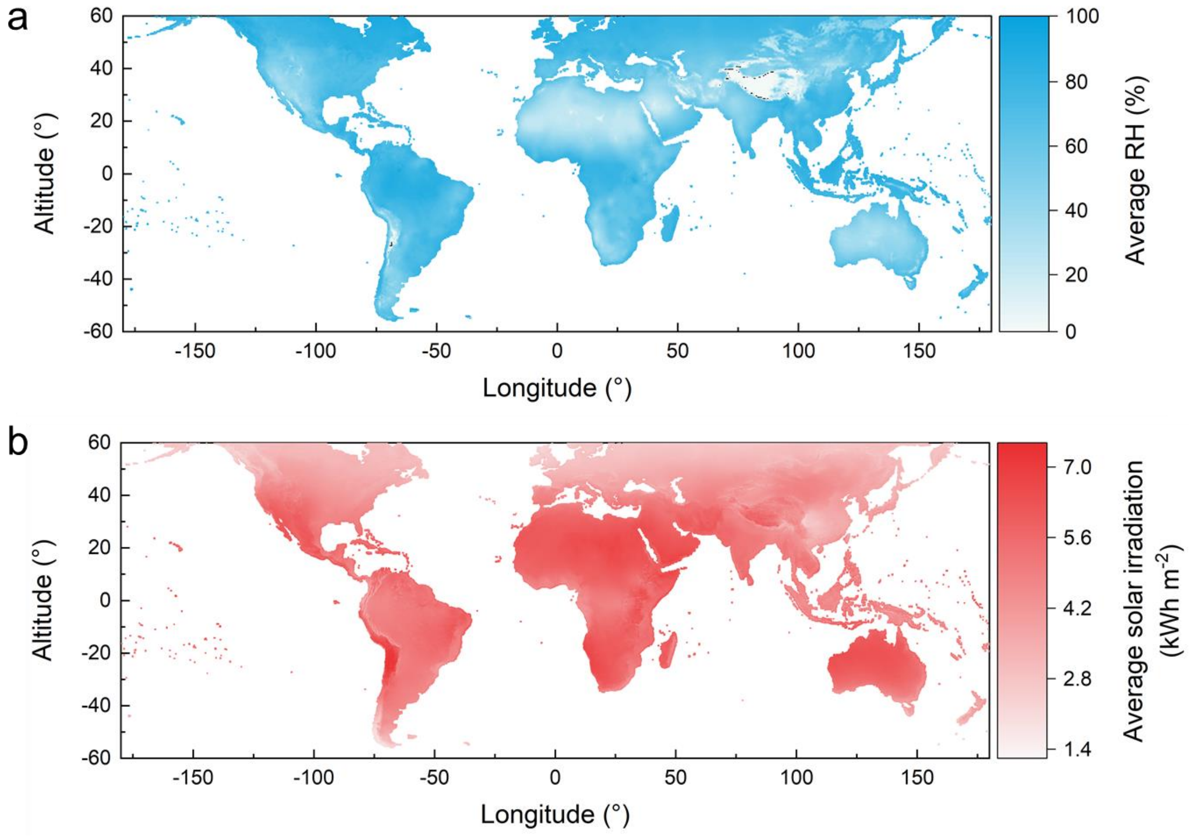

Supplementary Fig. 32| The yearly average of humidity and solar irradiation. a. The global yearly average RH condition. **b.** The global yearly average of solar irradiation.

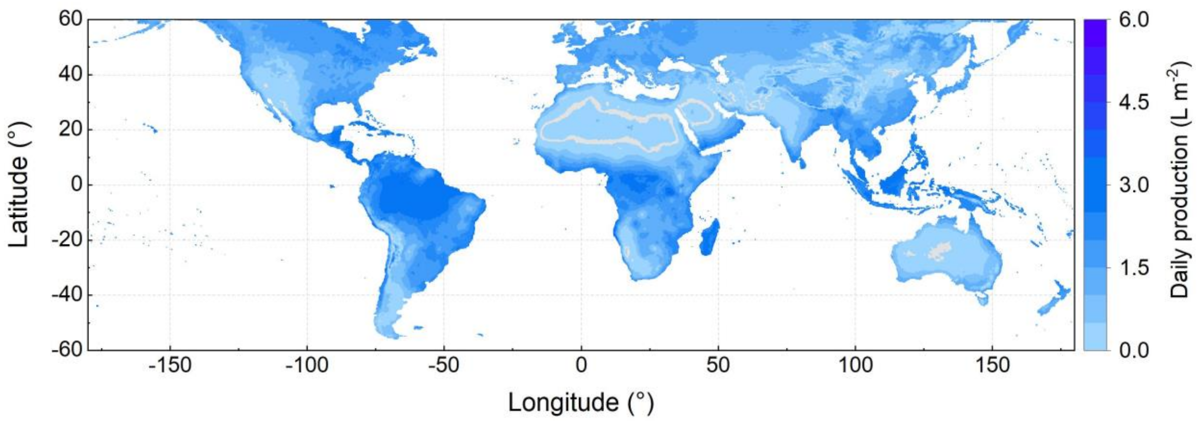

Supplementary Fig. 33| The global water production capability of the system working in manual mode.

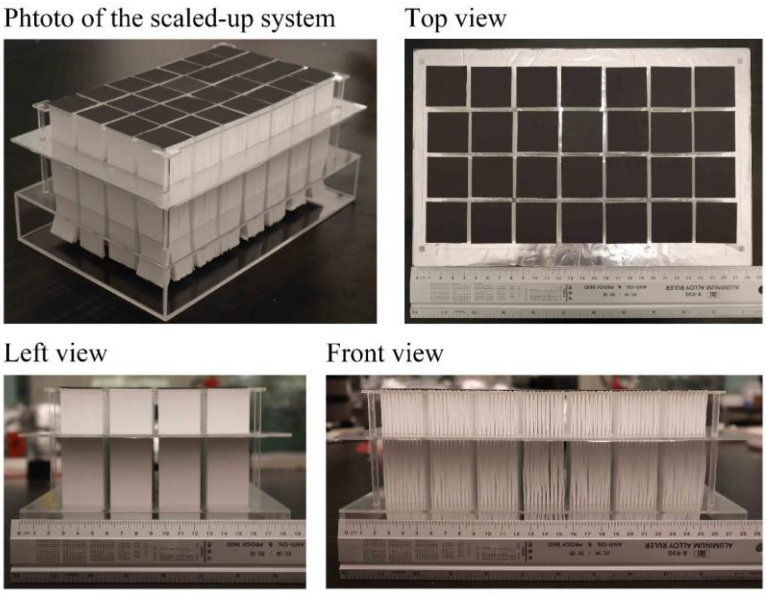

Supplementary Fig. 34| Photos of the scaled-up prototype.

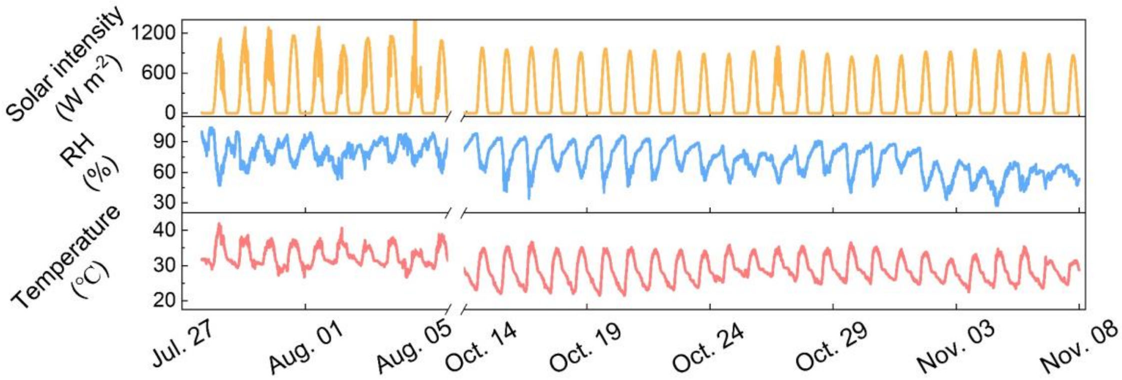

Supplementary Fig. 35| The real-time weather condition variation during the outdoor test.

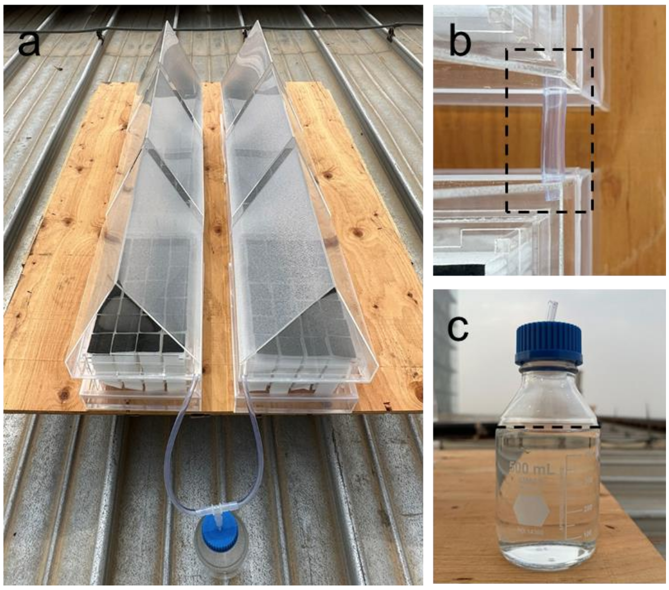

Supplementary Fig. 36| Scaled-up demonstration. a, A scaled-up system by connecting six prototypes. **b**, Connecting between each prototypes. **c**, Water generated from the scaled-up system. With an appropriate incline angle, generated water can flow through the connecting pipe and enter into the collection bottle. When six prototypes are connected in series, the scaled-up system (footprint: 23 cm × 93 cm) can produce ~480 ml of fresh water per day, which is approximately six times the productivity of each unit, indicating that the effect of mass transfer issues is negligible. The evaluation commenced at 6:00 pm on Sep. 7, 2023, and ended at 6:00 pm on Sep. 8, 2023.

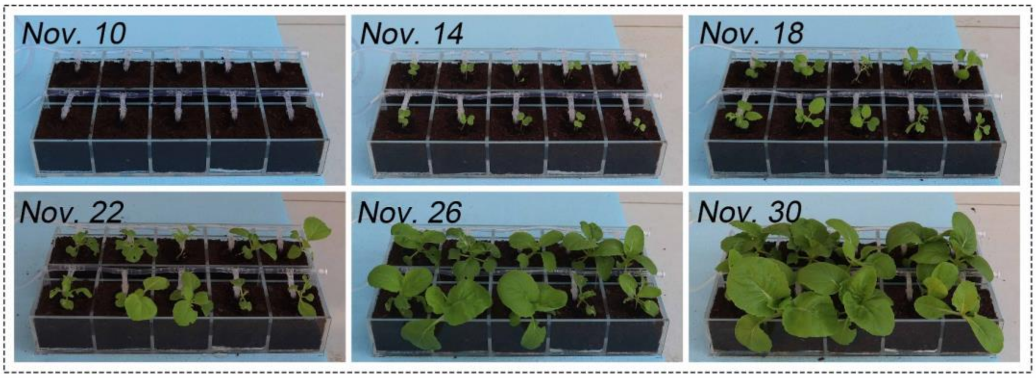

Supplementary Fig. 37| The growth of Chinese cabbage irrigated with tap water.

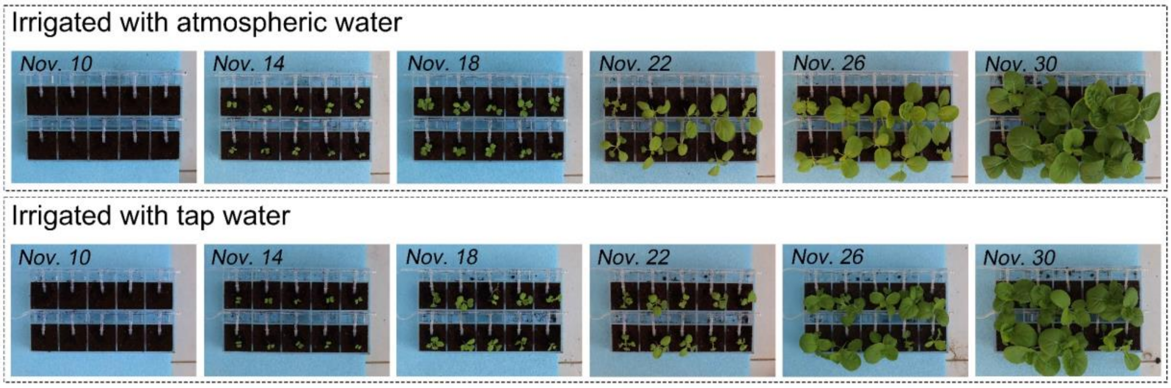

Supplementary Fig. 38| The photo recording of the plants growth from the top-view perspective.

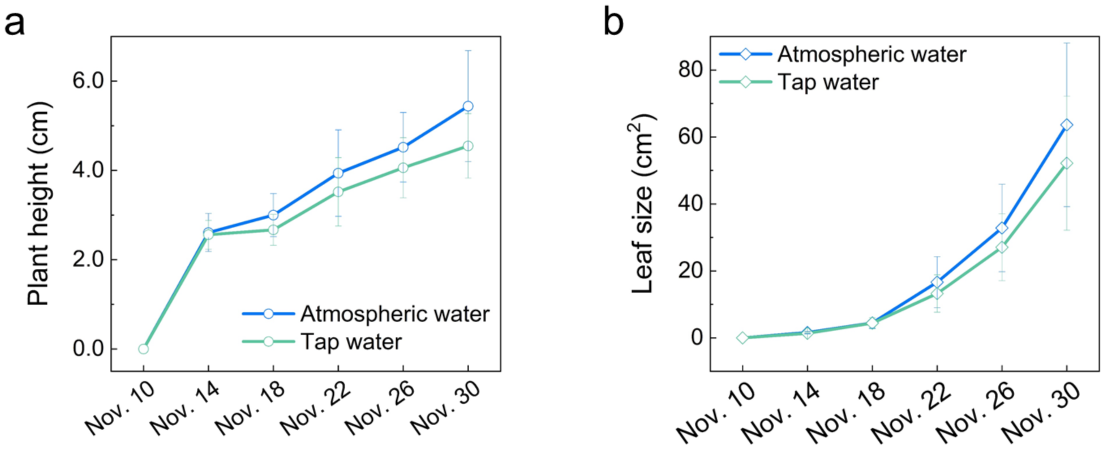

Supplementary Fig. 39| Plants growth characterization. a, The height variation of the plants irrigated with atmospheric water and tap water. **b,** The leaf size variation of plants irrigated with atmospheric water and tap water. (The error bar presents the standard deviation between 10 plants.)

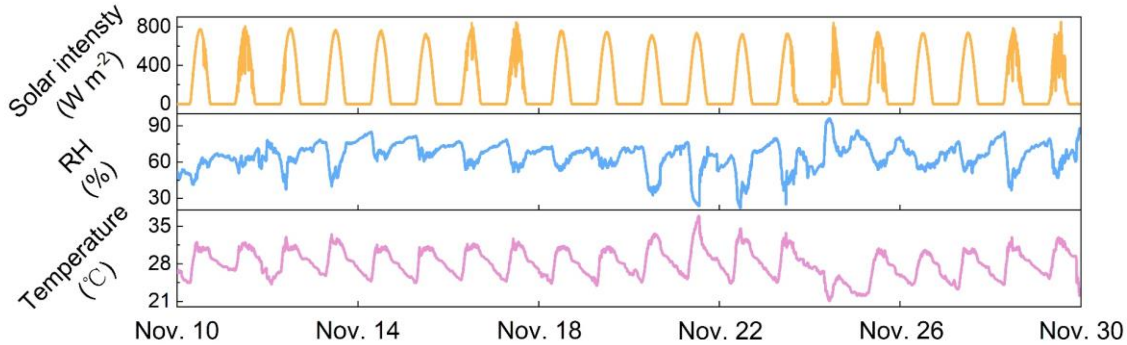

Supplementary Fig. 40| The real-time weather recording during plant growth.

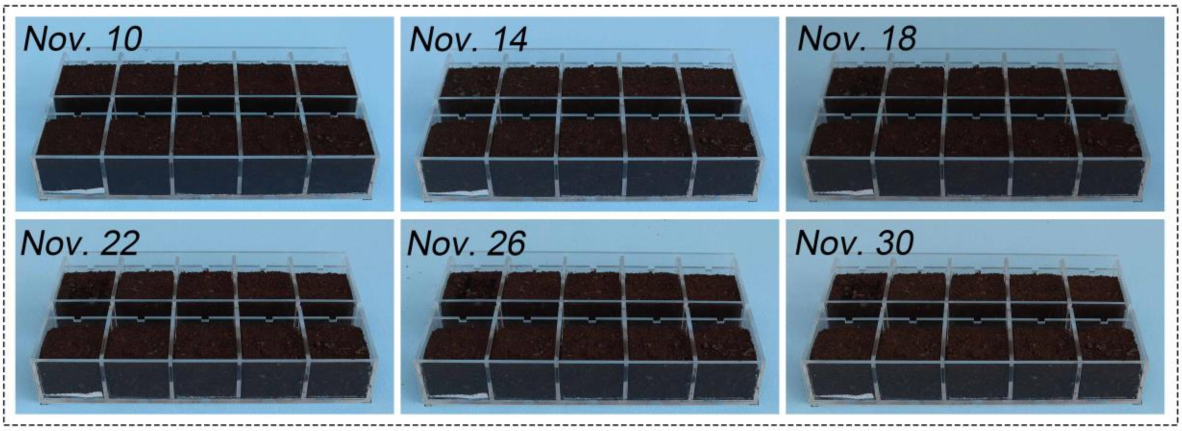

Supplementary Fig. 41| The growth of Chinese cabbage without irrigation.

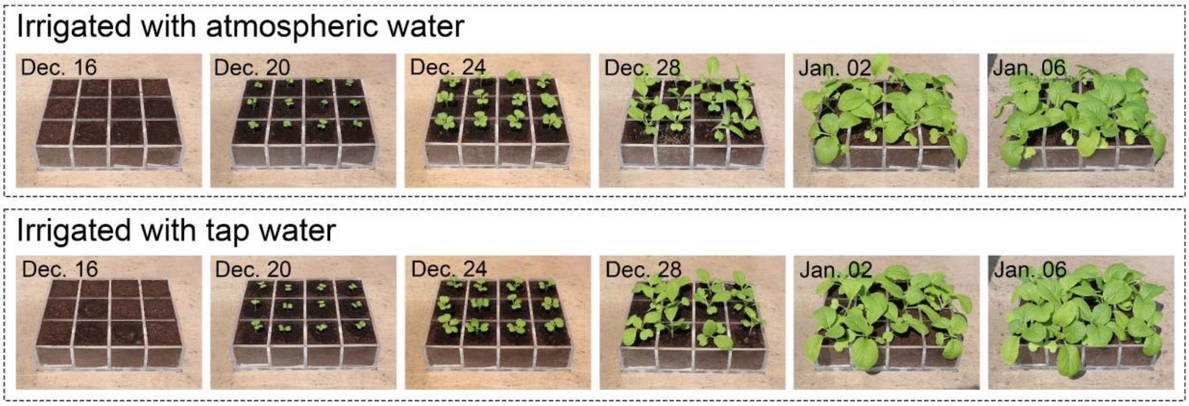

Supplementary Fig. 42| Repetition experiment on the rooftop in KAUST, demonstrating the growth progression of Chinese cabbage irrigated with atmospheric water and tap water.

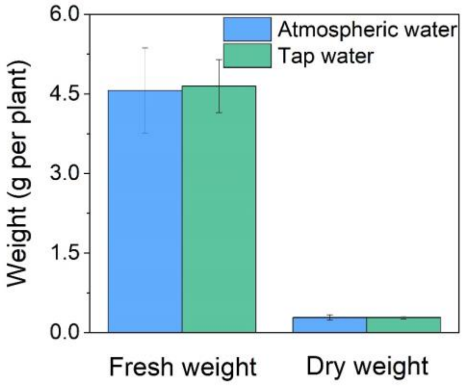

Supplementary Fig. 43| The fresh weight and dry weight of the harvested plants in the repetition experiment. (The error bar presents the standard deviation between 10 plants.)

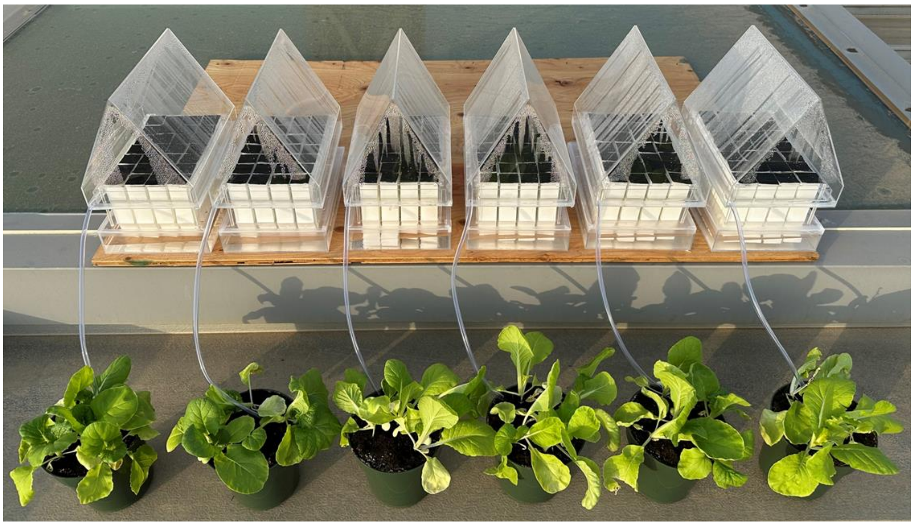

Supplementary Fig. 44| Demonstration of irrigation application by putting the prototype next to the plant and directing the generated water to the plant root.

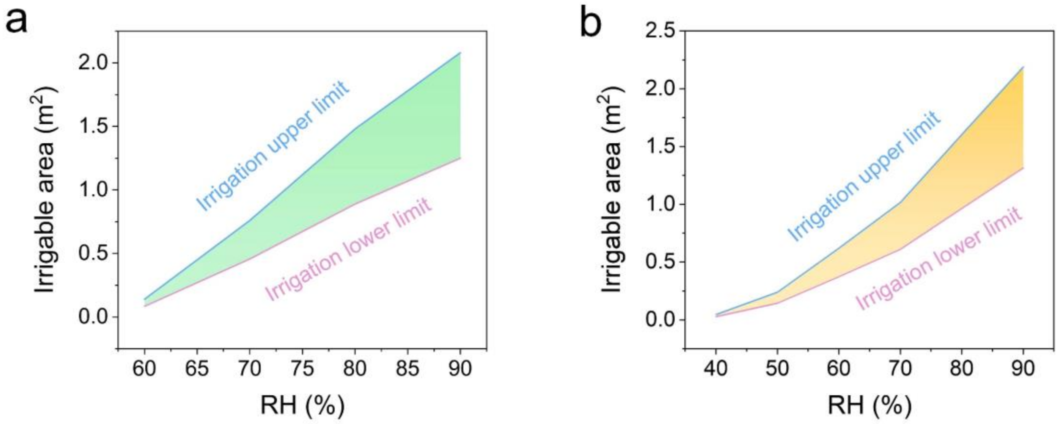

Supplementary Fig. 45| The irrigable area of Chinese cabbage that one square meter system can sustain. a, System operated in maintenance-free mode. **b**, System operated in manual operation mode.

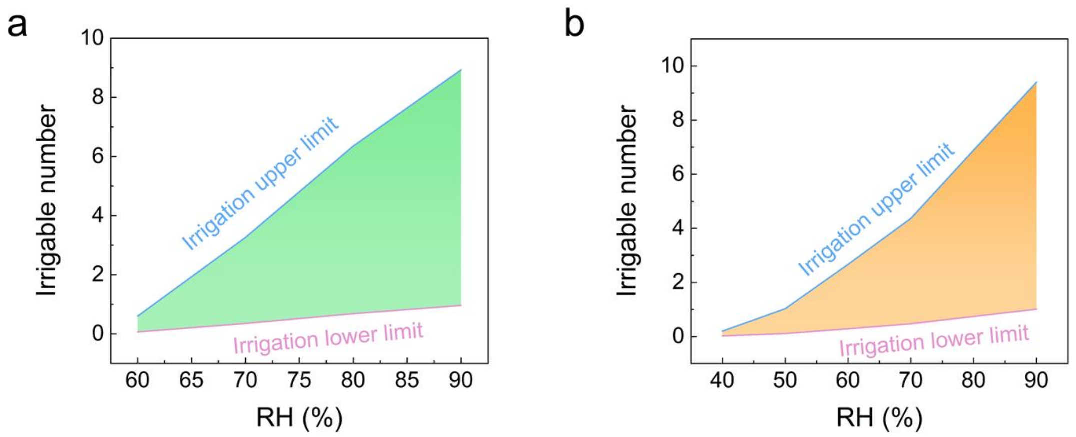

Supplementary Fig. 46| The irrigable area of *Vachellia tortilis* that one square meter system can sustain. a, System operated in maintenance-free mode. **b**, System operated in manual operation mode.

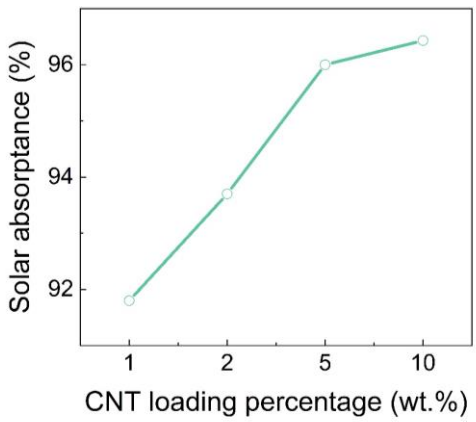

Supplementary Fig. 47| The influence of CNT loading percentage on solar absorptance.

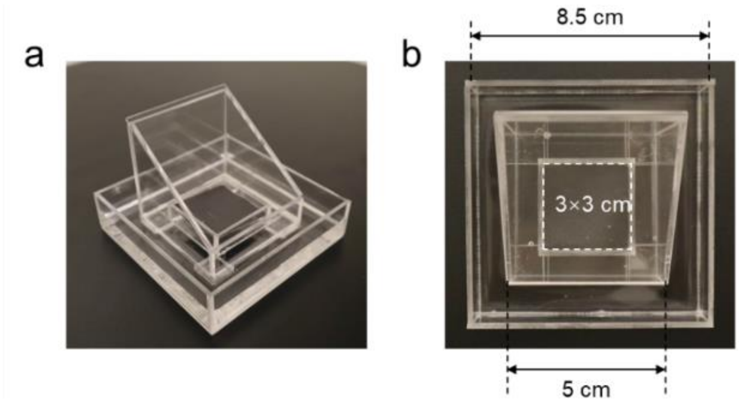

Supplementary Fig. 48| The system reported in the previous study of *Adv. Mater.* 31, e1903378, (2019). a, A photo of the fabricated system. **b**, The detailed size information of the fabricated.

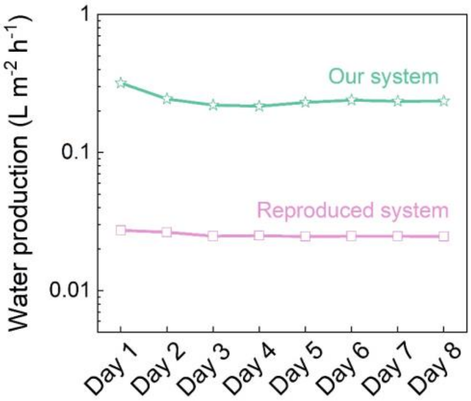

Supplementary Fig. 49| Performance comparison between the reported system and our system at 70% RH.

NO.	System structure	Materials	Operation	RH	Water production	Ref.
1	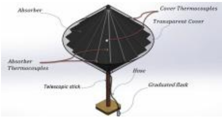	$CaCl_2$	Manual operation required	Not given	0.33 - 0.63 L m^{-2} h^{-1}	2
2	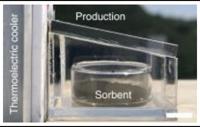	MOF-derived porous carbon	Manual operation required; Active cooler is used	26%	0.16 L kg^{-1} h^{-1}	3
3	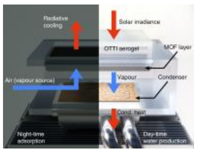	MOF-801	Manual operation required	40%	0.12 L kg^{-1} per day	4
4	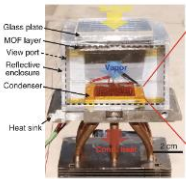	MOF-801	Manual operation required; Active cooler is used	20%	0.9 L m^{-2} per day	5
5	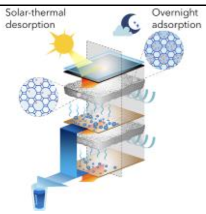	AQSOA Z01	Manual operation required	40%	0.77 L m^{-2} per day	6
6	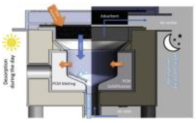	$LiCl/MgSO_4$/ACF	Manual operation required	35%	0.92 kg kg^{-1}	7
7	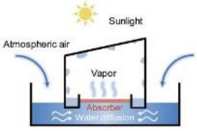	[EMIM][Ac]	Fully passive	70%	2.8 kg m^{-2} per day	8
8	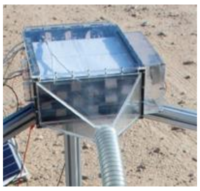	MOF-303	Electricity required	32%	1.3 L kg^{-1} per day	9
9	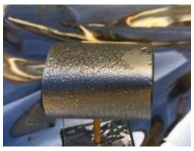	PDMS-based radiative cooling material	Fully passive	95%	~0.08 L m^{-2} h^{-1}	10

**10**	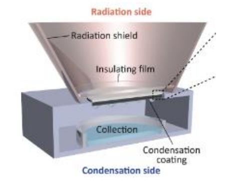	PDMS-based radiative cooling material	Fully passive	95%	0.059 L m^{-2} h^{-1}	11
**11**	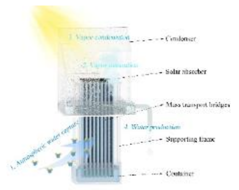	LiCl solution	Fully passive	60% - 90%	0.04 - 0.65 L m^{-2} h^{-1}	This study
			Manual operation is required	40% - 90%	0.02 - 0.68 L m^{-2} h^{-1}	

Supplementary Table 1| Comparison between the reported AWE devices. Images reprinted with permission: part **1** from ref. 2. Copyright (2018) Elsevier; part **2** from ref. 3, Springer Nature Limited; part **3** from ref. 4, Springer Nature Limited; part **4** from ref. 5, AAAS; part **5** from ref. 6, Copyright (2021) Elsevier; part **6** from ref. 7, Copyright (2020) Elsevier; part **7** from ref. 8. Wiley; part **8** from ref. 9. CC BY 4.0; part **9** from ref. 10, PNAS; part **10** from ref. 11, AAAS.

Supplementary Table 2| The microbial indicators of the collected water.

**Indicator**	**Concentration**
HPC 36oC	< detection limit
Coliforms	< detection limit
Escherichia coli	< detection limit
ATP	< detection limit

Supplementary Table 3| Raw data of the irrigated plants' weight.

	Irrigated with atmospheric water		Irrigated with tap water	
Plant number	Fresh weight (g)	Dry weight (g)	Fresh weight (g)	Dry weight (g)
1	3.4	0.19	2.2	0.12
2	6.6	0.37	1.7	0.10
3	4.6	0.26	6.4	0.36
4	7.5	0.42	5.6	0.31
5	5.2	0.29	4.0	0.22
6	3.7	0.21	4.6	0.26
7	6.7	0.37	4.9	0.28
8	4.3	0.24	3.2	0.18
9	6.7	0.37	5.3	0.30
10	1.9	0.11	3.7	0.21
Average weight (g)	5.06	0.28	4.16	0.23
standard deviation (g)	1.80	0.10	1.50	0.08

Supplementary Table 4| The daily water requirement for Chinese cabbage and *Vachellia tortilis* (Data from *Ref* 12 and *Ref* 13)**.**

Species	Daily water requirement
Chinese cabbage	2.50 – 4.16 L m^{-2} per day
Vachellia tortilis	0.14 – 1.3 L m^{-2} per each

Supplementary References

1. M. R. Conde, Properties of aqueous solutions of lithium and calcium chlorides: formulations for use in air conditioning equipment design. *Int. J. Therm. Sci.* **43**, 367–382, (2004)

2. M. A. Talaat, M. M. Awad, E. B. Zeidan and A. M. Hamed. Solar-powered portable apparatus for extracting water from air using desiccant solution. *Renew. Energ.* **119**, 662-674, (2018).

3. Y. Song, et al. High-yield solar-driven atmospheric water harvesting of metal-organic-framework-derived nanoporous carbon with fast-diffusion water channels. *Nat. Nanotechnol.* **17**, 857-863, (2022).

4. H. Kim, et al. Adsorption-based atmospheric water harvesting device for arid climates. *Nat. Commun.* **9**, 1191, (2018).

5. H. Kim, et al. Water harvesting from air with metal-organic frameworks powered by natural sunlight. *Science* **356**, 430–434, (2017).

6. A. LaPotin, et al. Dual-stage atmospheric water harvesting device for scalable solar-driven water production. *Joule* **5**, 166-182, (2021).

7. M. Ejeian, A. Entezari, R.Z. Wang, Solar powered atmospheric water harvesting with enhanced LiCl /$MgSO_4$/ACF composite. *Applied Thermal Engineering* **176**, 115396, (2020)

8. H. Qi, et al. An interfacial solar-driven atmospheric water generator based on a liquid sorbent with simultaneous adsorption-desorption. *Adv. Mater.* **31**, e1903378, (2019).

9. N. Hanikel, et al. Rapid cycling and exceptional yield in a metal-organic framework water harvester. *ACS Cent. Sci.* **5**, 1699-1706, (2019).

10. M. Zhou, et al. Vapor condensation with daytime radiative cooling. *Proc. Natl. Acad. Sci. U.S.A.* **118**, (2021).

11. I. Haechler, et al. Exploiting radiative cooling for uninterrupted 24-hour water harvesting from the atmosphere. *Sci. Adv.* **7**, eabf3978, (2021).

12. C. Brouwer and M. Heibloem, Irrigation Water Management: Irrigation Water Needs. *Food and Agriculture Organization of the United Nations*, (1986)

13. M. A. Al-Sinan, A. A. Bubshait and F. Alamri, Saudi Arabia's journey toward net-zero emissions: progress and challenges. *Energies*, **16**, 978 (2023).
