# Supplementary material for: A solar-driven atmospheric water extractor for off-grid freshwater generation and irrigation
Source: Nat Commun. 2024 Jul 24;15:6260. doi: 10.1038/s41467-024-50715-0 (PMC11269568; doi:10.1038/s41467-024-50715-0)
Supplement: Supplementary file 3 — Description of Additional Supplementary Files [file 41467_2024_50715_MOESM3_ESM.pdf]

Description of Additional Supplementary Files

File Name: Supplementary Movie 1
Description: A movie recording of the prototype's operation process, demonstrating its working process of atmospheric water capture and freshwater generation at 90% RH.

File Name: Supplementary Movie 2
Description: A movie recording of the real-time water production during field test. The test was performed on a rooftop at KAUST on July 27, 2022.
