# Supplementary material for: A solar-driven atmospheric water extractor for off-grid freshwater generation and irrigation
Source: Nat Commun. 2024 Jul 24;15:6260. doi: 10.1038/s41467-024-50715-0 (PMC11269568; doi:10.1038/s41467-024-50715-0)
Supplement: Supplementary file 6 — Reporting Summary [file 41467_2024_50715_MOESM6_ESM.pdf]

nature portfolio

Corresponding author(s): Qiaoqiang Gan; Yu Han

Last updated by author(s): Jul 10, 2024

Reporting Summary

Nature Portfolio wishes to improve the reproducibility of the work that we publish. This form provides structure for consistency and transparency in reporting. For further information on Nature Portfolio policies, see our Editorial Policies and the Editorial Policy Checklist.

Statistics

For all statistical analyses, confirm that the following items are present in the figure legend, table legend, main text, or Methods section.

n/a	Confirmed	
☐	☒	The exact sample size (*n*) for each experimental group/condition, given as a discrete number and unit of measurement
☐	☒	A statement on whether measurements were taken from distinct samples or whether the same sample was measured repeatedly
☒	☐	The statistical test(s) used AND whether they are one- or two-sided<br>*Only common tests should be described solely by name; describe more complex techniques in the Methods section.*
☒	☐	A description of all covariates tested
☒	☐	A description of any assumptions or corrections, such as tests of normality and adjustment for multiple comparisons
☒	☐	A full description of the statistical parameters including central tendency (e.g. means) or other basic estimates (e.g. regression coefficient) AND variation (e.g. standard deviation) or associated estimates of uncertainty (e.g. confidence intervals)
☒	☐	For null hypothesis testing, the test statistic (e.g. *F*, *t*, *r*) with confidence intervals, effect sizes, degrees of freedom and *P* value noted<br>*Give P values as exact values whenever suitable.*
☒	☐	For Bayesian analysis, information on the choice of priors and Markov chain Monte Carlo settings
☒	☐	For hierarchical and complex designs, identification of the appropriate level for tests and full reporting of outcomes
☒	☐	Estimates of effect sizes (e.g. Cohen's *d*, Pearson's *r*), indicating how they were calculated

Our web collection on statistics for biologists contains articles on many of the points above.

Software and code

Policy information about availability of computer code

Data collection	No computer code was used in this work.
Data analysis	No computer code was used in this work.

For manuscripts utilizing custom algorithms or software that are central to the research but not yet described in published literature, software must be made available to editors and reviewers. We strongly encourage code deposition in a community repository (e.g. GitHub). See the Nature Portfolio guidelines for submitting code & software for further information.

Data

Policy information about availability of data

All manuscripts must include a data availability statement. This statement should provide the following information, where applicable:

- Accession codes, unique identifiers, or web links for publicly available datasets
- A description of any restrictions on data availability
- For clinical datasets or third party data, please ensure that the statement adheres to our policy

Data availability
The authors declare that all data supporting this work are contained in graphics displayed in the main text or in the Supplementary Information. Data used to generate these figures are available from the authors upon request. Source data are provided with this paper.

Research involving human participants, their data, or biological material

Policy information about studies with human participants or human data. See also policy information about sex, gender (identity/presentation), and sexual orientation and race, ethnicity and racism.

Reporting on sex and gender	This research did not involve human participants, human data or biological material
Reporting on race, ethnicity, or other socially relevant groupings	This research did not involve human participants, human data or biological material
Population characteristics	This research did not involve human participants, human data or biological material
Recruitment	This research did not involve human participants, human data or biological material
Ethics oversight	This research did not involve human participants, human data or biological material

Note that full information on the approval of the study protocol must also be provided in the manuscript.

Field-specific reporting

Please select the one below that is the best fit for your research. If you are not sure, read the appropriate sections before making your selection.

☐ Life sciences ☐ Behavioural & social sciences ☒ Ecological, evolutionary & environmental sciences

For a reference copy of the document with all sections, see nature.com/documents/nr-reporting-summary-flat.pdf

Ecological, evolutionary & environmental sciences study design

All studies must disclose on these points even when the disclosure is negative.

Study description	This study demonstrate a fully passive solar-driven SAWE system for atmospheric water harvesting and irrigation.
Research sample	The research subject of this work is the newly designed AWH system. We focus on its water production capacity and its potential applications in irrigation.
Sampling strategy	Sampling test was not involved

Data collection

Performance evaluation

The performances of prototypes were evaluated in a research chamber (AR66L, Percival) where the temperature and RH can be controlled accurately. The evaluations were carried out at 60% RH, 25℃ with the MTBs structure of the prototypes initially infiltrated with 0.24 g/g LiCl solution. For water production performance evaluation, the vapor generation zone was enclosed in the condenser chamber and the water production rate was determined by measuring the collected water. For evaporation performance evaluation, the condenser chamber was removed to allow for vapor release, the atmospheric water capture zone was sealed to avoid the effect of water capture, and the evaporation rate was determined by detecting the weight change. For water capture performance evaluation, the atmospheric water capture zone was exposed to the environment while the vapor generation zone was enclosed, and the water uptake rate was determined by measuring the weight change. To evaluate the performance of water capture and water production at different RH and different LiCl concentration, the environment RH and the infiltrated LiCl solution concentration was regulated accordingly.

The experiment, which involved recording the system's operational process using time-lapse photography, was conducted at 90% RH, 25℃ with MTBs structure initially infiltrated with 0.24 g/g LiCl solution. The prototype underwent 12 h of water capture without sunlight, followed by 12 h of water production under 1 sun illumination.

The simultaneous water capture and production performance was evaluated at 65% RH, 25℃ with MTBs structure initially infiltrated with 0.24 g/g LiCl solution. The water capture performance was determined by recording the weight change of the prototype and the water production was measured by weighting the condensed water.

The day-by-day cycling evaluation was performed to evaluate the water production potential under different RH conditions. The MTBs structure of the prototype was infiltrated with 0.24 g/g LiCl and the temperature was controlled at 25℃. In each cycle, the prototype first underwent 16h of water capture followed by 8h water production under 1 sun illumination. The water production rate was calculated according to the condensed water in the chamber.

Field tests

The outdoor experiment was conducted on the rooftop in KAUST. The environmental conditions, including RH, temperature, and solar intensity were recorded by a weather station (HP2550, Misol). The solar energy was calculated according to the following equation: $E=A\times\int P(t)dt$ where E (kJ) represents the received solar energy, A (m2) is the irradiated area, P (kW/m2) is the solar intensity and t (s) is the time. The average daily temperature and humidity are calculated based on the real-time weather data between 19:00 yesterday to 19:00 today. The produced water was collected by a graduated cylinder and the process was recorded by a camera. The ions concentration was measured by ICP-OES (5110, Agilent). The total intact cell concentration was quantified by flow cytometry (BD Accuri C6, Belgium). HPC was determined using the Quanti-Tray method. Colilert-18 test kit (IDEXX, USA) was used to simultaneously detect total coliforms and E. coli in the water sample. The active biomass present in the water samples were evaluated with a luminometer (Celsis Advance Luminometer, Charles River Laboratories). Specifically, LuminEx and LuminATE-HS were used to release the ATP and produce light.

The off-grid irrigation experiment was performed on the balcony in KAUST. A custom-built acrylic tray with ten individual compartments (6×6×5 cm) was used for plant growth. Chinese cabbage seed (Quality Cabbage, Longda Seed) was selected for demonstration and standard potting soil (Basissubstrat 2, Stender) was used for plant growth. The automatic drip irrigation was controlled by a timing pump which supplied 9 ml of water to each compartment daily at 20:00. The volumetric water content and the conductivity of the soil were detected by a soil detector (PR-3001-TRREC-N01, Presens) every night at 19:00. The probe was located 3 cm away from the roots to avoid any interference on the plant growth. The plant growth was recorded by a camera at 8:00. The plants height was measured with a ruler. The leaf number was accounted every 4 days. Specifically, the leaf size was determined by tracing its outline on a grid paper and calculating the area of the outline. We place the leaf on the grid paper with squares that are 0.25 cm2 in area. Then, we traced the outline of the leaf on the grid paper and counted the number of the squares to get the surface of each leaf. The plants were harvested after 20 days of growth. The wet biomass of plants was recorded after cleaning the soil attached to the roots, and the dry biomass was determined following dehydration at 80℃ for 12h. The repetition experiment of plant irrigation was performed on the rooftop in KAUST. This repetition contains two sets of plant growth, one was irrigated with the collected atmospheric water and the other was irrigated with the tap water. The irrigation water volume for each plant (i.e. ~9 ml) and watering time (i.e. at 20:00 each day) were kept consistent with the previous experiment. The plant growth was recorded accordingly. The fresh weight and the dry weight of the harvested plants were analyzed.

Timing and spatial scale	The outdoor experiment was conducted on the rooftop in KAUST. The filed test of water production was performed form Jul. 27, 2022 to Aug. 6, 2022 and from Oct. 14, 2022 to Nov. 8, 2022.
Data exclusions	No date was excluded
Reproducibility	All lab experiments were repeated 3 times to check the data reproducibility. For plant growth experiment, 10 plants were used to confirm the feasibility.
Randomization	Laboratory measurements were conducted under controlled conditions, data almost has no randomization. Field test result is related to the environment condition which was recorded in real-time by weather station and was presented in the main text and supplementary information.
Blinding	The data was measured by laboratory equipment and there is no blinding during acquisition and analysis.

Did the study involve field work? ☒ Yes ☐ No

Field work, collection and transport

Field conditions	The filed test started from Jul. 27, 2022 to Aug. 6, 2022 and from Oct. 14, 2022 to Nov. 8, 2022. Weather condition variation was recorded and presented in the main text and supplementary information.
Location	Jeddah, Saudi Arabia
Access & import/export	This research involves on habitats access.
Disturbance	The field test causes no disturbance.

Reporting for specific materials, systems and methods

We require information from authors about some types of materials, experimental systems and methods used in many studies. Here, indicate whether each material, system or method listed is relevant to your study. If you are not sure if a list item applies to your research, read the appropriate section before selecting a response.

Materials & experimental systems

n/a	Involved in the study	
☒	☐	Antibodies
☒	☐	Eukaryotic cell lines
☒	☐	Palaeontology and archaeology
☒	☐	Animals and other organisms
☒	☐	Clinical data
☒	☐	Dual use research of concern
☐	☒	Plants

Methods

n/a	Involved in the study	
☒	☐	ChIP-seq
☒	☐	Flow cytometry
☒	☐	MRI-based neuroimaging

Dual use research of concern

Policy information about dual use research of concern

Hazards

Could the accidental, deliberate or reckless misuse of agents or technologies generated in the work, or the application of information presented in the manuscript, pose a threat to:

No	Yes	
☒	☐	Public health
☒	☐	National security
☒	☐	Crops and/or livestock
☒	☐	Ecosystems
☒	☐	Any other significant area

Experiments of concern

Does the work involve any of these experiments of concern:

No	Yes	
☒	☐	Demonstrate how to render a vaccine ineffective
☒	☐	Confer resistance to therapeutically useful antibiotics or antiviral agents
☒	☐	Enhance the virulence of a pathogen or render a nonpathogen virulent
☒	☐	Increase transmissibility of a pathogen
☒	☐	Alter the host range of a pathogen
☒	☐	Enable evasion of diagnostic/detection modalities
☒	☐	Enable the weaponization of a biological agent or toxin
☒	☐	Any other potentially harmful combination of experiments and agents

Plants

Seed stocks	In this work, Chinese cabbage seed was selected for demonstration, which is commercially available. It was purchased from Longda Seed and stocked at 25℃
Novel plant genotypes	This plant has no novel plant genotypes.
Authentication	The Chinese cabbage seed we used is commercially available, which was purchased from Longda Seed and stored under dry conditin with a temperature ~25℃
